# Expression of geminiviral AC2 RNA silencing suppressor changes sugar and jasmonate responsive gene expression in transgenic tobacco plants

**DOI:** 10.1186/1471-2229-12-204

**Published:** 2012-11-07

**Authors:** Arto J Soitamo, Balaji Jada, Kirsi Lehto

**Affiliations:** 1 Department of Biochemistry and Food Chemistry, Laboratory of Molecular Plant Biology, University of Turku, Vesilinnantie 5, LTII, 2.floor, Turku, 20014, Finland

## Abstract

**Background:**

RNA-silencing is a conserved gene regulation and surveillance machinery, which in plants, is also used as major defence mechanism against viruses. Various virus-specific dsRNA structures are recognized by the silencing machinery leading to degradation of the viral RNAs or, as in case of begomoviruses, to methylation of their DNA genomes. Viruses produce specific RNA silencing suppressor (RSS) proteins to prevent these host defence mechanisms, and as these interfere with the silencing machinery they also disturb the endogenous silencing reactions. In this paper, we describe how expression of AC2 RSS, derived from *African cassava mosaic geminivirus* changes transcription profile in tobacco (*Nicotiana tabacum*) leaves and in flowers.

**Results:**

Expression of AC2 RSS in transgenic tobacco plants induced clear phenotypic changes both in leaves and in flowers. Transcriptomes of these plants were strongly altered, with total of 1118 and 251 differentially expressed genes in leaves and flowers, respectively. The three most up-regulated transcript groups were related to stress, cell wall modifications and signalling, whereas the three most down-regulated groups were related to translation, photosynthesis and transcription. It appears that many of the gene expression alterations appeared to be related to enhanced biosynthesis of jasmonate and ethylene, and consequent enhancement of the genes and pathways that are regulated by these hormones, or to the retrograde signalling caused by the reduced photosynthetic activity and sugar metabolism. Comparison of these results to a previous transcriptional profiling of HC-Pro RSS-expressing plants revealed that some of same genes were induced by both RSSs, but their expression levels were typically higher in AC2 than in HC-Pro RSS expressing plants. All in all, a large number of transcript alterations were found to be specific to each of the RSS expressing transgenic plants.

**Conclusions:**

AC2 RSS in transgenic tobacco plants interferes with the silencing machinery. It causes stress and defence reactions for instance via induction of the jasmonate and ethylene biosynthesis, and by consequent gene expression alteration regulated by these hormones. The changed sugar metabolism may cause significant down-regulation of genes encoding ribosomal proteins, thus reducing the general translation level.

## Background

### Gene silencing in plants

The conserved molecular machinery of RNA-silencing constitutes a very complex genetic regulatory network in all eukaryotes. The common features for these regulatory pathways are their induction by double stranded (ds) RNA sequences. In plants, these are cleaved by the RNAse III type DICER-LIKE (DCL) enzymes, assisted by HYPONASTI LEAVES1 (HYL1), SERRATE (SE) and DAWLE (DLL) proteins [1] into 21–25 nucleotide long RNAs, called either (micro-) miRNAs or (short interfering) siRNA. Upon cleavage, the short RNA fragments are methylated at their 3’ ends by the S-adenosyl methionine dependent methyltransferase HEN1 [2], and loaded into effector complexes, called RITC (RNA induced transcription silencing complex) or transported to cytoplasm apparently with the assistance of HASTY protein [3] and loaded RISC (RNA induced silencing complex) reviewed in [4]. The RITS complexes contain siRNA, AGO4 and AGO6 to mediate transcriptional silencing (TGS) to repetitive or over-expressed DNA sequences via histone and DNA methylation, leading to heterochromatin maintenance, control of transposon mobility and transgene silencing. This RNA-dependent DNA methylation (RdDM) machinery utilizes at least the DNA-directed RNA polymerases PolIV and PolV, RNA-dependent RNA polymerase 2 (RDR2), Dicer-like 3 (DCL3) enzyme, AGO4, AGO5, AGO6 and DNA methyltransferase (DRM2) [5], chromatin-remodelling protein (DRD1), structural-maintenance-of-chromosomes-protein (DMS3) and RdDM effector molecule KTF1, which binds scaffold transcripts generated by PolV and recruits AGO4 bound siRNAs to form an RdDM effector complexes [6,7].

The RISC complexes, targeted to RNA sequences, cause the post-transcriptional silencing (PTGS) of the target mRNAs either via their cleavage or translational arrest [8]. MiRNA-mediated PTGS is used, for instance, to regulate the temporal and spatial expression of multiple different transcription factors, many of which are needed in the developmental differentiation of plant organs, and also regulate various components of the silencing pathways, thus back-regulating these pathways themselves. In addition, numerous miRNAs, as well as a plethora of various siRNAs are known to be involved in genetic responses and signalling cascades induced by various hormones and biotic and abiotic stresses (reviewed in [9-13]).

The small RNAs target these silencing complexes to partially or fully complementary DNA or RNA sequences, respectively. On these target molecules the effector complexes mediate multiple essential regulatory functions like transcriptional gene silencing (TGS) or post-transcriptional gene silencing (PTGS) or decreasing the translational rate (reviewed by [8,14]). In plants RNA silencing is used as the major defence mechanism against viruses.

### Suppression of RNA Silencing in plants

To counteract the silencing mediated host defence, plant viruses encode various viral RSSs [15]. Functional suppression of RNA silencing appears to be required for any virus to successfully invade and accumulate in its host plant, and several of the RSSs have been identified as essential viral pathogenicity factors and symptom determinants already before their function in RNA silencing suppression was discovered [16]. It is of interest that the RSS factors encoded by different viral families have no similarities with each other, and many viruses produce their RSS activity as a secondary function of some gene product that has also some other function in viral life cycle, e.g. in replication, cell-to cell or systemic movement or encapsulation (reviewed in [17]). The high complexity of the silencing machinery provides multiple steps where it can be disturbed, and the mechanisms of the different RSS are very diverse. The hosts’ silencing machineries and their molecular interactions with viral RSSs have been intensively studied over the last decade [8,10,17-20] and they appear to be excellent tools for interrupting and analysing of the RNA silencing pathways.

### Begomoviruses and silencing suppression in plants

Begomoviruses, with circular ssDNA genomes, produce transcripts in opposite orientation. They form partly dsRNA structures and activate plant’s RNA silencing machinery, but can resist this suppression by producing active RSSs [21-23]. The silencing is not (at least totally) targeted against viral RNA transcripts, but rather against the viral replicative intermediate, i.e. the minichromosome structures formed of replicative viral dsDNA, combined with histone proteins [21]. The silencing is mediated by the TGS, i.e. by methylation of these viral dsDNA sequences, which leads to repression of their replication and transcription [24]. Total of three different types of RSS are known to be encoded by *African cassava mosaic* geminivirus (ACMV), which nowadays is classified as a begomovirus [23,25]. The main RSS has been identified as the transcriptional activator protein AC2, encoded in opposite sense of the begomoviral minichromosome.

Here we have analysed the transcriptome and proteome of the transgenic tobacco plants expressing the AC2 RSS, derived from ACMV begomovirus. Alterations detected in these gene expression profiles were compared to the those that we have earlier detected in similar transgenic tobacco plants that express the HC-Pro RSS derived from *Potato virus Y potyvirus*[26], revealing the fundamental similarities and differences between the functional mechanisms of these two RSS in transgenic tobacco plants.

## Results

### Phenotype of tobacco plants expressing AC2 RSS

**Figure 1:**
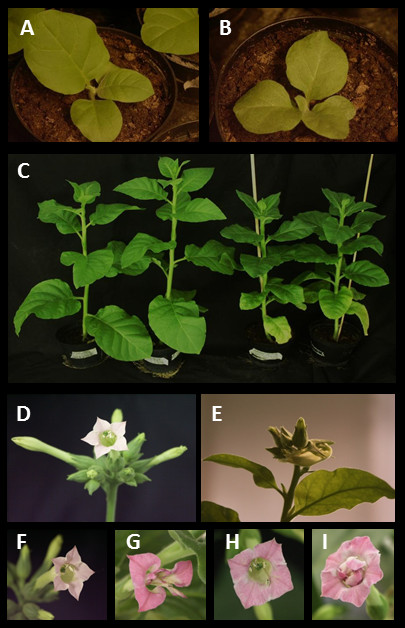
**Phenotypic changes in AC2 expressing transgenic tobacco plants.** Three week-old wild type (**A**) and AC2 expressing tobacco plants (**B**). Two biological replicates of six-week old wild type (on the left) and AC2 expressing tobacco plants (on the right) (**C**). Unopened and opened flowers of wild type tobacco plants (**D** and **F**) and of AC2 expressing tobacco plants (**E**, **G**, **H** and **I**).

A previously characterized transgenic tobacco line expressing Geminiviral AC2 RSS under a constitutive CaMV 35S promoter was used in this study [27]. Transgenic tobacco plants expressing AC2 RSS (Additional file 1) were grown concurrently under the same growth conditions that were earlier used for HC-Pro expressing transgenic tobacco plants [26]. Expression of nuclear AC2 protein in transgenic tobacco plants induced clear phenotypic changes both in leaves and flowers, including different malformations of veins and leaves of the young transgenic plants (Figure 1A and 1B). These malformations gradually disappeared as plants become older, but there still were some phenotypic changes in six week-old leaves; the leaves were narrower and paler green than leaves in wild type plants (Figure 1C). The growth habit was stubby. Lower leaves were often yellow, the root neck and root system was less developed than in wild type plants, and mature plants were slightly smaller in size compared to wild type tobacco plants. Flower buds were sometimes twisted round other flower buds (Figure 1E). The structure of flowers were often, but not always drastically malformed; petals were grown together having either round or more often a rectangular shape. Stamen filaments were also often changed to extra petals (Figure 1G- 1I).

### Experimental design and differential gene expression in AC2 expressing plants

The gene expression profiling of the AC2 RSS expressing plants was performed at the same time as that of the HC-Pro expressing transgenic tobacco plants by using the same wild type and empty vector (pBIN61) expressing control plants as were used for the transgenic HC-Pro expressing plants, using three biological replicates for each. The intensity values indicating differential gene expression between control and the AC2 expressing plants were normalized using the Chipster program (CSC, Espoo, Finland). The intensity values originating from the leaf and flower samples were normalized separately as was performed for the HC-Pro samples [26], (Additional file 2). Two-fold change in the expression between AC2 transgenic and control samples was regarded as significant level of differential expression. Statistical significance of differentially expressed genes was tested using Students t-test, p values less than 0.05 with False Discovery Rate (FDR) (See Additional file 3 Additional file 4 Additional file 5 Additional file 6). Microarray results were verified by using RT-qPCR. with a good correlation between RT-qPCR and array results both in up- and –down-regulated transcripts, and both in leaves and flowers (Table 1). Transcripts, whose expression did not change, were used as reference transcripts [28].

**Table 1:** Verification of microarray results using RT-qPCR

		**Microarray**	**RT-qPCR**	
**EST/mRNA**	**Description**	**Fold**	**Fold**	**s.e**
**Leaf (up-regulated transcripts**				
EH615198	Nicotiana tabacum, nictaba (NT1) mRNA Jasmonic acid methyl ester and ethylene-induced mRNA	27.7	22.3	4.9
EB438380	Solanum lycopersicum, Trypsin and protease inhibitor, mRNA	13.7	10.1	2.9
FG156808	Nicotiana tabacum, 1-D-deoxyxylulose 5-phosphate synthase (DXS) mRNA	3.0	2.7	0.8
**Leaf (down-regulated transcript)**				
AY741503	Actin binding protein 1L; ABIL-1L	0.6	0.2	0.11
**Leaf (non-regulated transcripts)**				
X67159	Nicotiana tabacum pectate lyase mRNA	0.97	0.9	0.04
**Flower (up-regulated transcripts)**				
EB683763	Nicotiana tabacum EIG-29C mRNA	2.95	2.95	0.8
EB438380	Solanum lycopersicum Trypsin and protease inhibitor, mRNA	2.55	2.55	0.67
**Flower (down-regulated transcripts)**				
X65700	Nicotiana tabacum osmotin, AP-24 mRNA	0.2	0.2	0.5
**Flower (non-regulated transcripts)**				
NP917355	Nicotiana tabacum mRNA for ERF1	1.51	1.51	0.28

Some clearly up- or down-regulated genes of leaf and flower samples were tested. Statistical significance was tested using Student’s t-test (p <0.05).Fold change is indicated as a ratio of AC2/WT calculated from normalized median intensity values (n=3). Standard error of mean (s.e.) is also calculated for RT-qPCR values.

The numbers of differentially expressed transcripts, assigned to various functional groups, in leaves and flowers of transgenic AC2 expressing plants is presented in Table 2. The total number of altered transcripts was 1118 and 251 in leaves and flowers, respectively. In leaves total of 726 transcripts were up-regulated and 392 down-regulated, with the three biggest groups of up-regulated transcripts being related to signalling, cell wall modifications, and stress, whereas the three most down-regulated groups of transcripts being related to protein synthesis, photosynthesis and transcriptional regulation. The results demonstrated in Table 2 were based on counting of genes in functional categories similarly as it was performed in our previous publication [26] but not the overpresentation analysis.

**Table 2:** An overview of microarray results demonstrating differentially expressed genes in leaves and flowers in AC2 expressing plants

**Functional characterization *expression of genes***	**AC2 leaf *(UP)***	**AC2 leaf *(DOWN)***	**AC2 flower *(UP)***	**AC2 *flower *(DOWN)**
Defence related	38	10	10	7
ROI related	33	5	3	3
Kinases and phosphatases	26	11	0	4
Transcriptional regulators	46	**26**	8	10
Protein degradation and proteases	25	5	2	8
Lipases and hydrolases	19	1	1	4
Transporters	39	8	3	8
HSPs	8	6	1	3
Signalling	**66**	7	7	2
Cell wall related	**63**	17	7	13
Stress related	**50**	14	8	5
Protein synthesis related	10	**67**	2	1
Photosynthesis related	31	**53**	5	0
RNA binding	8	10	1	0
Interesting miscellaneous	111	60	18	41
Unknown function	153	92	30	36
**Total**	**726**	**392**	**106**	**145**

The table represents number of genes that were up or down regulated more than two-fold. Statistical significance was tested by using paired t-test (p <0.05).

**Figure 2:**
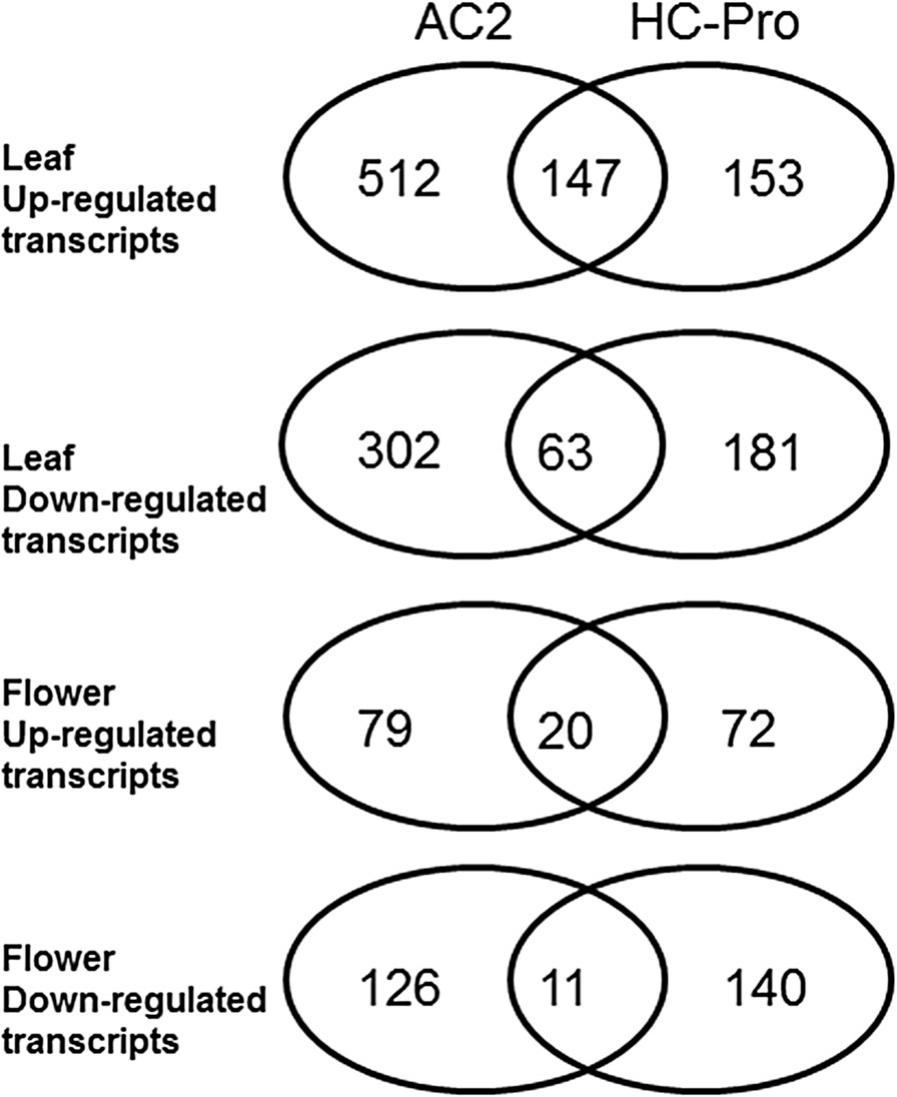
**A Venn-diagram presenting number of up –and down regulated transcripts between AC2 and HC-Pro expressing transgenic tobacco plants.** Data was presented after removal of duplicated samples.

About half of the transcripts up-regulated in leaves of the HC-Pro expressing plants were found up-regulated also in the leaves of the transgenic AC2 expressing plants (Figure 2), but more than five hundred additional transcripts were up-regulated only in the AC2 expressing plants. The same pattern was also found in the down-regulated transcripts of the leaves; more than three hundred transcripts were down-regulated only in AC2 expressing plants. The number of differentially expressed transcripts was much lower in flowers but these also included common and specific transcripts between AC2 and HC-Pro expressing plants (Figure 2). See also Additional file 7 for over and under presentation of functional categories between HC-Pro and AC2 RSS expressing plants.

### Expression of AC2 in tobacco plants induces clear defence responses

**Figure 3:**
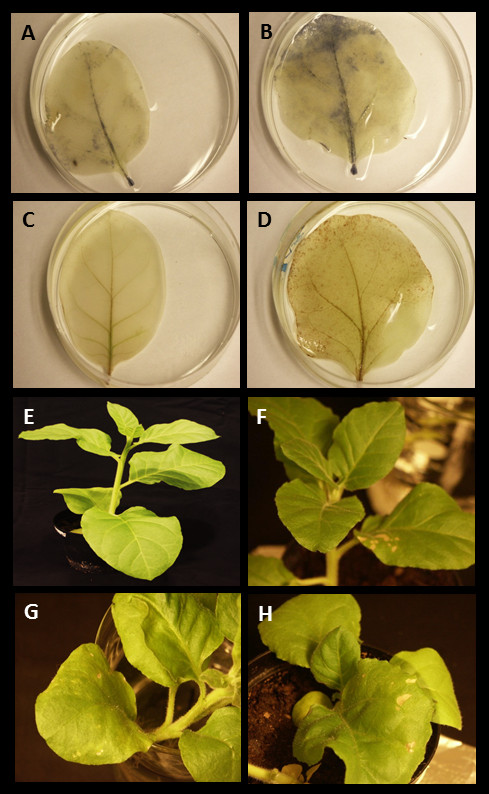
**Determination of oxidative stress (A-D) and hypersensitive reaction (E-H) in wild type and AC2 expressing transgenic leaves.** Superoxide radicals were determined using NBT chemical of one-month old wild type (**A**) and AC2 expressing (**B**) leaves and hydroperoxide radicals were determined using DAB chemical of one-month old leaves of wild type (**C**) and AC2 expressing (**D**). One-month old wild type (**E**) is presented as control plant and three AC2 expressing plants (**F-H**) that were showing hypersensitive reactions in lower leaves.

The expression of AC2 RSS in transgenic plants induced many defence- and oxidative stress- related transcripts, including transcripts for systemic acquired resistance (SAR), ROS scavenging and respiratory burst oxidase (Table 3, Additional file 8) which were not detected in HC-Pro RSS expressing transgenic plants [26]. The amount of oxidative stress was further studied using NBT staining for superoxide radical and DAB for H_2_O_2_ to indicate the amount different ROSes in leaves of AC2 expressing plants. The amount of ROSes was found increased only in one-month old AC2 expressing tobacco plants (Figure 3), while the amount of ROS accumulation in older and more developed leaves was similar as in wild-type leaves (data not shown).

**Table 3:** Defence, and jasmonic acid related up-regulated transcripts in AC2 expressing tobacco leaves

**Defense response**			**Jasmonate biosynthesis and signaling**		
EST/mRNA	Fold	Gene description	EST/mRNA	Fold	Gene description
TA16340_4097	8.5	Medicago truncatula Annexin	FG638546	3.8	Nicotiana tabacum PLA2 protein
FG636567	5.1	Nicotiana tabacum NtEIG-C29	FG638546	4.1	Nicotiana tabacum PLA2 protein
TA11690_4097	4.1	Nicotiana tabacum SAR8.2d protein	CV020743	3.7	Nicotiana tabacum PLA2 protein
AY775034	3.9	Nicotiana tabacum Avr9/Cf-9 protein	CV017220	2.0	Nicotiana tabacum PLA2 protein
TA11684_4097	3.9	Nicotiana tabacum Sar8.2c	EB681141	4.5	Nicotiana attenuata lipoxygenase
EH622972	3.8	Nicotiana tabacum Sar8.2j	DR752068	4.3	Nicotiana attenuata lipoxygenase
EB440996	3.8	Pathogenesis-related protein	EB430793	3.7	Nicotiana attenuata lipoxygenase
EH621322	3.6	Nicotiana tabacum SAR8.2e protein	EB447101	2.9	Nicotiana attenuata lipoxygenase
EH622818	3.3	Nicotiana tabacum: SAR8.2c protein	DV160344	2.1	Nicotiana attenuata lipoxygenase
EH619416	2.5	Capsicum annuum Defensin J1-2	FG644491	8.0	Solanum tuberosum allene oxide syntase
TA14524_4097	2.4	Nicotiana tabacumAvr9/Cf-9 protein 65	TA15530_4097	2.6	Solanum lycopersicum 12-oxophytodienoate reductase 3
EH620366	2.2	Nicotiana benthamiana Respiratory burst oxidase	TA15531_4097	2.0	Solanum lycopersicum 12-oxophytodienoate reductase 3
CV018796	2.1	SAR8.2m protein related cluster	DW004249	2.0	Nicotiana tabacum Allene oxide cyclase
BP132210	2.1	Populus trichocarpa TIR-NBS disease resistance-like protein	AJ308487	2.4	Nicotiana tabacum Allene oxide cyclase
TA18382_4097	2.0	Avr9/Cf-9 rapidly elicited protein 14	AJ308487	2.5	Nicotiana tabacum Allene oxide cyclase
**Scavenging of reactive oxygen species (ROS)**			EB427874	2.4	Nicotiana tabacum NtJAZ1 protein
DW000487	14.7	Glutaredoxin-C13	EB448804	2.7	Nicotiana tabacum NtJAZ1 protein
AY074787	4.7	Nicotiana tabacum Dehydroascorbate reductase	TA15051_4097	3.4	Nicotiana tabacum NtJAZ1 protein
FG637828	2.8	Nicotiana tabacum peroxidase	EB681065	2.0	Nicotiana tabacum NtJAZ1 protein
TA12995_4097	2.7	Nicotiana sylvestris phospholipid hydroperoxide glutathione peroxidase	EB445549	2.4	Nicotiana tabacum NtJAZ3 protein
EH622305	2.7	Glutathione S-transferase	EB427874	2.9	TIFY protein of group II and jasmonic acid-related stress response
EB439671	2.7	Nicotiana alata Thioredoxin H	CV016937	2.7	TIFY protein of group II and jasmonic acid-related stress response
FG143852	2.1	Solanum tuberosum peroxidase (prx2)	**Jasmonic acid responsive genes**		
CV018765	2.1	Nicotiana tabacum thioredoxin	EH615998	27.7	Nicotiana tabacum nictaba (NT1)
			EH615198	23.4	Nicotiana tabacum nictaba (NT1)
EF532799	2.1	Nicotiana tabacum catalase	EB444740	5.6	Nicotiana glutinosa 1-aminocyclo-propane-1-carboxylic acid oxidase (ACC oxidase)
			FG633784	5.2	Solanum lycopersicum anthocyanin acyltransferase
			NP917355	4.9	Nicotiana tabacum ERF1

Fold change is indicated as a ratio of AC2/WT calculated from normalized median intensity values (n = 3).

Interestingly, hypersensitive reaction (HR, or necrotic lesions) was frequently detected in lower leaves of two month old transgenic AC2 expressing plants (Figure 3F- 3H). These kinds of necrotic lesions were never detected in leaves of wild type tobaccos of similar age. This suggested that particularly the young developing leaves suffered from oxidative stress where also the enhanced levels of ROS-related transcripts were detected.

### Jasmonic acid biosynthesis and jasmonate-responsive genes

Multiple genes related to jasmonic acid (JA) biosynthesis were up-regulated in leaves of transgenic AC2 expressing plants. Some of these genes were also up-regulated in HC-Pro expressing plants, but in these the level of up-regulation was much lower [26]. Also genes encoding jasmonic acid-mediated regulatory proteins like NtJAZ1 and NtJAZ3 repressors were up-regulated (Table 3, Additional file 7). The presence of jasmonates (e.g.JA-Ile) induces proteasomal degradation of these JAZ repressors and thereby activates transcription e.g. from promoters containing MYC binding sites (reviewed in [29]). Many of the JA-responsive genes e.g. defence and stress-related transcripts were also up-regulated. Jasmonic acid signalling cascade also positively regulates the genes involved in biosynthesis of jasmonic acid itself (from chloroplast 18:3 fatty acids to JA) [29,30], and in the biosynthesis of ethylene [31,32]. Table 3 shows almost full induction of jasmonic acid biosynthesis related transcripts, jasmonic acid regulatory genes as well as some well-known jasmonic acid responsive genes, including the 1-aminocyclopropane-1-carboxylic acid oxidase (ACC oxidase) transcript, required for the biosynthesis of ethylene from its ACC precursor [31,32] in leaves of transgenic AC2 expressing plants. In addition, a transcript of ethylene response factor 1 (ERF1), a transcription factor responsible for mediating ethylene responsive gene expression [32] was up-regulated about five-fold.

### Chlorophyll biosynthesis and photosynthesis related genes were down–regulated in AC2 expressing transgenic plants

Most of the phenotypic changes occurred in young, less than two-month old plants, but not many differences were detected in older leaves of AC2 expressing plants. Leaves of young transgenic plants (up to two-month old) were more yellow than those of the wild type tobaccos, indicating changes in their pigment content. The pigment analysis revealed that the amount of both chlorophyll a and b were decreased in them (Additional file 9), and there was also a clear decrease in the accumulation of anthocyanin in the same plants, as compared to wild type plants. The decreased amount of chlorophyll in leaves was not surprising since many of the genes related to chlorophyll biosynthesis were clearly down-regulated (Table 4). Table 4 also depicts down-regulation of transcripts of photosystem II and I related genes, as well transcripts related to primary carbon metabolism.

**Table 4:** Down-regulated chloroplast targeted transcripts in AC2 expressing tobacco leaves

**EST/mRNA**	**Fold**	**Gene description**	**EST/mRNA**	**Fold**	**Gene description**
**Chlorophyll synthesis**			**Photosystem II**		
FG152482	0.27	Arabidopsis thaliana uroporphyrinogen decarboxylase (HEME1)	TA17489_4097	0.47	Arabidopsis thaliana thylakoid lumenal 29.8 kDa protein
BP528863	0.30	Arabidopsis thaliana uroporphyrinogen decarboxylase (HEME1)	DW000743	0.48	S.oleracea 6.1 kDa polypeptide of photosystem II
FG636717	0.34	Tomato phytoene synthetase	DV162613	0.49	Arabidopsis thaliana thylakoid lumenal 29.8 kDa protein
EB435329	0.36	Medicago truncatula Porphobilinogen deaminase	AY220076	0.50	Nicotiana tabacum Oxygen evolving complex 33 kDa photosystem II protein
TA14322_4097	0.36	Nicotiana tabacum Mg protoporphyrin IX chelatase	**Photosystem I**		
EB435329	0.38	Medicago truncatula Porphobilinogen deaminase	EB439123	0.44	Nicotiana tabacum chloroplast ferredoxin I (fdn-1)
BP134714	0.39	Ricinus communis uroporphyrinogen decarboxylase	EB681816	0.46	Nicotiana attenuata photosystem I subunit XI
EB680805	0.43	Arabidopsis thaliana non-yellowing protein 1 (NYE1),	TA15418_4097	0.49	cytochrome b6f complex assembly protein
CV017484	0.44	Nicotiana tabacum POR1 NADPH: protochlorophyllide oxidoreductase	**Primary carbom metabolism**		
CV018932	0.44	Nicotiana tabacum POR1 NADPH: protochlorophyllide oxidoreductase	TA11967_4097	6.31	Nicotiana sylvestris Rubisco large subunit
X82833	0.45	Nicotianatabacum uroporphyrinogen decarboxylase	TA21396_4097	0.18	Nicotiana tabacum Rubisco activase 1,
			FG135141	0.38	S.tuberosum cytosolic fructose-1,6-biphosphatase
DW001114	0.45	Ricinus communis chlorophyll synthase	TA19588_4097	0.41	Medicago truncatula Aldo/keto reductase
DV999032	0.46	Solanum lycopersicum cultivar Red Setter phytoene synthase 1 (psy1)	DW000256	0.42	Arabidopsis thaliana CHUP1 (chloroplast unusual positioning 1)
FG133694	0.46	Arabidopsis thaliana HEMD uroporphyrinogen-III synthase (HEMD)	CV018672	0.45	Ricinus communis rubisco subunit binding-protein alpha subunit
M29868	0.48	Ricinus communis uroporphyrin-III methyltransferase	EB680256	0.46	Solanum tuberosum granule-bound starch synthase (GBSS)
DV161248	0.48	Ricinus communis uroporphyrin-III methyltransferase	BP532702	0.46	Ricinus communis malate dehydrogenase
TA14111_4097	0.48	Capsicum annuum Phytoene synthase	TA14326_4097	0.48	Nicotiana tabacum Nucleoside diphosphate kinase 2, chloroplast
**Chloroplast fatty acid synthesis**			TA11656_4097	0.49	Solanum tuberosum plastidic aldolases
TA16177_4097	0.28	Arabidopsis thaliana Palmitoyl-monogalactosyldiacylglycerol delta-7 desaturase	**Chloroplast ribosomal RNA**		
TA15510_4097	0.38	Olea europaea Chloroplast fatty acid desaturase 6	TA11649_4097	0.33	Nicotiana tabacum chloroplast 16S ribosomal RNA-23S ribosomal RNA
DW001145	0.41	Olea europaea Chloroplast fatty acid desaturase 6	**Chloroplast ribosomal protein**		
BP129232	0.42	Nicotiana tabacum digalactosyl-diacylglycerol synthase (dgd1)	TA13552_4097	0.44	Arabidopsis thaliana 50S ribosomal protein L15
AJ632923	0.49	Nicotiana tabacum digalactosyl-diacylglycerol synthase (dgd1)	EB679716	0.46	Nicotiana tabacum (clone: L24-1) chloroplast ribosomal protein L24
			TA15069_4097	0.40	Arabidopsis thaliana 30S ribosomal protein S9, chloroplast precursor -
			TA20218_4097	0.44	Arabidopsis thaliana 30s ribosomal protein s1
			DV999348	0.45	Ricinus communis Plastid-specific 30S ribosomal protein
			TA15068_4097	0.48	Arabidopsis thaliana 30S ribosomal protein S9, chloroplast
			TA13672_4097	0.48	Spinacia oleracea 30S ribosomal protein S5, chloroplast precursor
			TA13755_4097	0.34	Medicago truncatula Ribosomal protein S

Fold change is indicated as a ratio of AC2/WT calculated from normalized median intensity values (n = 3).

**Figure 4:**
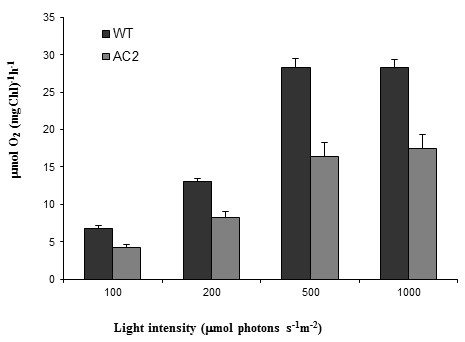
**Photosynthetic oxygen evolution of thylakoids isolated from leaves of wild type and AC2 expressing plants.** Oxygen evolution was measured at four different light intensities. Dark columns are indicating oxygen evolution of wild type thylakoids and lighter columns oxygen evolution of AC2 expressing thylakoids. Standard error of mean is presented above the columns (n = 6).

**Figure 5:**
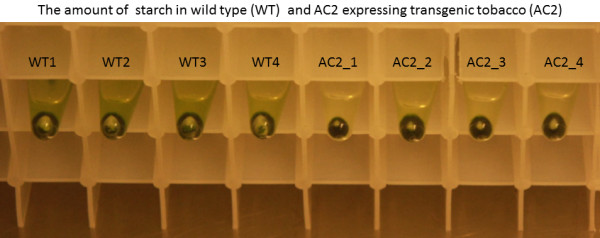
**Starch granules at the bottom of Eppenforf tube pelleted during thylakoid preparation.** For each of thylakoid isolation, 1 g of wild type (WT) or transgenic AC2 (AC2) leaves (fresh weight, FW) was used. Four biological replicates are presented in the figure.

Since there were clear changes in the genes encoding photosynthetic machinery proteins, the light-responsive oxygen evolution was measured from the thylakoid samples isolated from AC2 expressing and wild type leaves. Both photosynthetic oxygen evolution in freshly isolated thylakoids and the amount of starch were significantly decreased in transgenic AC2 expressing leaves, as compared to wild type tobacco leaves (Figures 4 and 5). The results were similar, but clearer than those published earlier for HC-Pro RSS expressing plants [26].

### Down-regulated accumulation of translation factor and ribosomal protein transcripts

**Figure 6:**
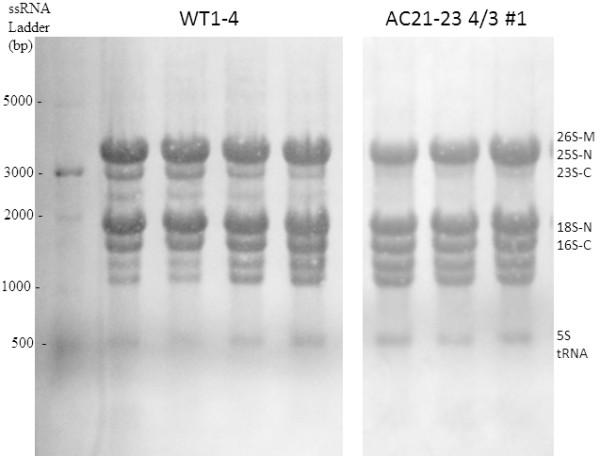
**The qualitative differences of ribosomal RNA in rRNA isolated from the wild type (WT) and AC2 expressing (AC2) transgenic tobacco leaves.** 10 μg of ribosomal RNA was separated in 1.2% TBE agarose gel. ssRNA ladder is shown in the left. Every lane corresponds to a biological replicate.

**Figure 7:**
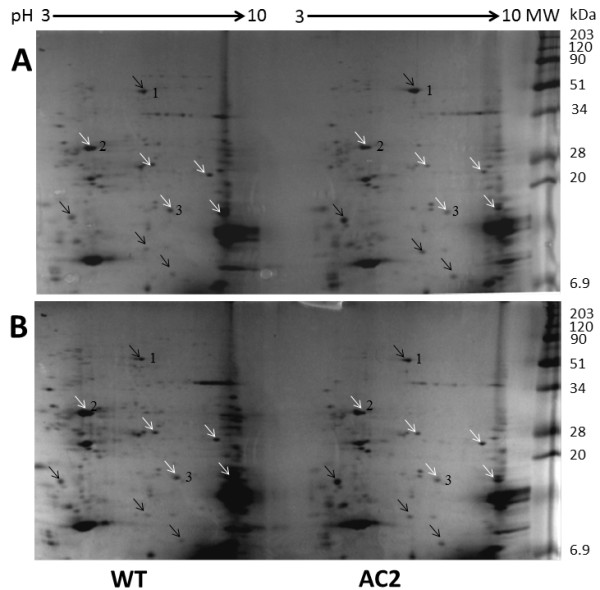
**Proteomic analysis of total protein samples isolated of wild type (WT) and AC2 expressing (AC2) transgenic tobacco leaves using 2D-SDS-PAGE.** Proteins separated in the first dimension by isoelectric focusing were separated in another dimension by SDS-PAGE. Both wild type and AC2 expressing total protein samples were run in the second dimension in a large SDS-polyacrylamide gel. Black arrows indicate up-regulated protein spots and white arrows down-regulated protein spots. Numbers indicate previously identified protein spots by mass spectroscopic methods (1, RBCL; 2, OEE33 and 3, CYP2) [26]. Two representative 2D-SDS-PAGE results are shown.

One of the most dramatic changes in the transcriptome of the transgenic AC2 expressing plants was the down regulation of genes encoding for translation factors and ribosomal protein subunits (Table 1 and Table 5, Additional file 7). Total of fifty two transcripts related to translation machinery, including genes encoding for the cytosolic large (60S) and small (40S) ribosomal protein subunits as well as chloroplast ribosomal protein subunits (50S and 30S), were down-regulated. There were also changes in the distribution of ribosomal RNAs (Figure 6), as the amount and ratio of 23S rRNA was clearly decreased in AC2 expressing tobacco plants. Consequently, the amount of total protein, as measured against fresh weight was decreased about 30% in AC2 expressing leaves compared to wild type tobacco leaves (Additional file 10). The proteomes of wild type and AC2 expressing tobacco leaves were compared by loading equal amounts of protein extracts (250μg) into the 2D-SDS-PAGE analysis (Figure 7), meaning that the analysis actually enhanced the protein spot intensities of the AC2-plant samples, as their total protein content was really lower than in the wild type plants. The AC2-plant proteome contained multiple changes, as compared to the wild type plant sample. To our surprise, the changes occurring in the proteomes of AC2 and HC-Pro expressing tobacco plants were quite similar to those observed in the HC-Pro expressing plants [26], although in general more proteins were down-regulated in AC2 expressing than in and HC-Pro expressing tobacco leaf.

**Table 5:** Genes encoding translation factor and ribosomal protein were down-regulated, whereas ribosomal RNA transcripts are up-regulated in transgenic AC2 expressing tobacco leaves

**EST/mRNA**	**Fold**	**Gene description**	**EST/mRNA**	**Fold**	**Gene description**
**Translation factors**			**Ribosomal protein (60S)**		
TA22209_4097	0.43	Nicotiana tabacum Eukaryotic translation initiation factor 3	TA14313_4097	0.45	Nicotiana tabacum L19 ribosomal protein L19
EB437355	0.35	Ricinus communis eukaryotic translation initiation factor 3	L27089	0.42	Petunia x hybrida ribosomal protein L15
DW000860	0.32	Glycine max Elongation factor G, chloroplast precursor	TA11947_4097	0.40	Nicotiana tabacum|Rep: Ribosomal protein L11-like
FG642632	0.25	Arabidopsis thaliana elongation factor P (EF-P)	EH622574	0.45	Nicotiana tabacum ribosomal protein L11
FG198791	0.31	Arabidopsis thaliana SCO1 translation elongation factor	EH619918	0.47	Nicotiana tabacum|Rep: Ribosomal protein L11-like
FG198791	0.35	Ricinus communis translation elongation factor G,	TA13020_4097	0.50	Ribosomal protein S11
EB439252	0.48	Solanum tuberosum ribosome-associated protein p40-like	CV015940	0.42	Ricinus communis ribosomal protein L5
TA16219_4097	0.41	Medicago truncatula Ribosome-binding factor A	FG152141	0.46	Nicotiana tabacum ribosomal protein L3A (RPL3A)
AM746200	0.40	Nicotiana tabacum glutamyl tRNA Reductase	**Ribosomal protein (40S)**		
BP135506	0.48	Ricinus communis lysyl-tRNA synthetase	FG640934	0.27	Zea mays 40S ribosomal protein S21
**Ribosomal protein (60S)**			EB678225	0.34	Solanum tuberosum 40S ribosomal protein S15
EH622574	0.29	Solanum tuberosum 60S ribosomal protein L21- like protein	TA12310_4097	0.45	Solanum tuberosum 40S ribosomal protein S19-like
DV161940	0.35	Lupinus luteus 60S ribosomal protein L30	DW001420	0.45	Arabidopsis thaliana 40S ribosomal protein S24
CV016994	0.36	Nicotiana tabacum (TSC40-3) 60S ribosomal protein L34	TA18231_4097	0.46	Solanum tuberosum|Rep: Putative 40S ribosomal protein S8
TA14313_4097	0.38	Solanum 60S ribosomal protein L38	DV159788	0.46	Capsicum annuum 40S ribosomal protein S2
CV021750	0.43	Arabidopsis thaliana 60S ribosomal protein L22-2	EB678225	0.47	Solanum tuberosum clone 40S ribosomal protein S15
DW000198	0.43	N.tabacum RL2 60S ribosomal protein L2	TA12310_4097	0.48	Solanum tuberosum 40S ribosomal protein S19
TA12097_4097	0.44	Solanum lycopersicum 60S ribosomal protein L8	EH615086	0.48	Ricinus communis 40S ribosomal protein S8
CV018461	0.45	Nicotiana tabacum (TSC40-4) 60S ribosomal protein L34 mRNA	FG142425	0.49	Ricinus communis 40S ribosomal protein S2
TA15366_4097	0.47	Solanum lycopersicum Similar to 60S ribosomal protein L35	FG642795	0.49	Arabidopsis thaliana 40S ribosomal protein S20 (RPS20B)
TA13593_4097	0.48	Arabidopsis thaliana 60S ribosomal protein L41	TA12892_4097	0.49	Solanum demissum 40S ribosomal protein S9
TA12097_4097	0.48	Solanum lycopersicum 60S ribosomal protein L8	TA13535_4097	0.49	Solanum tuberosum 40S ribosomal protein S4
TA14313_4097	0.48	Solanum|Rep: 60S ribosomal protein L38	**Ribosomal RNA**		
L27089	0.48	Nicotiana tabacum (TSC40-3) 60S ribosomal protein	TA19036_4097	9.62	Nicotiana tabacum 18S ribosomal RNA gene, nuclear
TA11947_4097	0.50	Capsicum annuum 60S ribosomal protein L19	TA12851_4097	6.62	Coffea arabica 26S ribosomal RNA gene, mitochondrial
EH622574	0.50	Solanum tuberosum 60S ribosomal protein L21- like protein	TA17274_4097	4.28	Nicotiana sylvestris 5.8S rRNA gene
EH619918	0.47	Solanum tuberosum clone 106F12 ribosomal protein L38	TA19143_4097	3.78	Nicotiana tabacum 18S ribosomal RNA gene, nuclear
TA13020_4097	0.50	Glycine max Ribosomal protein L37	TA16088_4097	3.17	Olpidium brassicae 28S ribosomal RNA gene, nuclear 25S rRNA
CV015940	0.47	Nicotiana glutinosa ribosomal protein L31	TA11649_4097	0.33	Nicotiana tabacum chloroplast 16S 23S ribosomal RNA gene
FG152141	0.48	Solanum tuberosum ribosomal protein L27a-like			
DV157967	0.47	Nicotiana tabacum ribosomal protein L25			
TA12097_4097	0.34	Medicago truncatula Ribosomal protein L24E			

Fold change is indicated as a ratio of AC2/WT calculated from normalized median intensity values (n = 3).

### Expression of silencing related transcripts

Interestingly, the microarray data indicated that some silencing related transcripts involved in the DNA methylation were altered (Table 6). Two of the up-regulated transcripts (KTF1 and AGO5) have been described to function in RNA-directed DNA methylation [33,34]. Based on its ability to bind 24nt small RNAs, AGO5 could influence small viral RNA-directed DNA methylation (RdDM). The AGO5 has an effect on CG motifs rather than C residues in a CNG or CNN context. Counteracting the increased DNA methylation by these gene products, also Repressor of Silencing1 (NtROS1), functioning as a negative regulator of silencing was up-regulated. In addition, two chromatin methyl transferases and a structural maintenance of chromosomes, also suggested to function in *de novo* DNA methylation, were down-regulated.

**Table 6:** Expression of RNA silencing related transcripts in AC2 expressing tobacco leaves

**EST/mRNA**	**Fold**	**Gene description**	**EST/mRNA**	**Fold**	**Gene description**
FG163572	2.7	Arabidopsis thaliana AGO5 (ARGONAUTE 5)	TA12909_4097	0.27	Histone 3
DW002999	2.6	Arabidopsis thaliana KTF1	CV017977	0.33	Histone H3 related
EB432235	2.3	Nicotiana tabacum NtROS1, repressor of silencing 1,	DW003379	0.36	Histone 3 Camellia sinensis
FG147325	2.2	Ricinus communis N-acetyltransferase, putative, mRNA	FG636738	0.36	Ricinus communis histone H2A,
EB683859	2.1	Medicago truncatula GCN5-related N-acetyltransferase	FG636738	0.37	Ricinus communis histone H2AmRNA
TA19898_4097	0.49	Structural maintenance of chromosomes protein 4	DW003245	0.39	Histone 3 [Camellia sinensis
TA15035_4097	0.46	Chromomethylase-like protein Nicotiana tabacum|	FG637321	0.40	Nicotiana tabacum H3 histone,
			EB449808	0.41	Populus trichocarpa histone 2
			EB678867	0.43	Arabidopsis thaliana histone H3
			CV017209	0.43	Capsicum annuum histone H4 mRNA
			FG641470	0.43	Arabidopsis thaliana histone H3
			BQ843162	0.43	Solanum melongena histone H4-like protein
			FG645490	0.44	Arabidopsis thaliana histone H3 like protein
			FG638902	0.44	N.tabacum mRNA for histone H2B1
			FG645490	0.45	Arabidopsis lyrata histone H3, mRNA
			CV019874	0.45	Nicotiana tabacum histone H4
			DW004009	0.46	Nicotiana tabacum H2A histone
			CV019731	0.46	Histone H2A related cluster

Fold change is indicated as a ratio of AC2/WT calculated from normalized median intensity values (n = 3).

**Figure 8:**
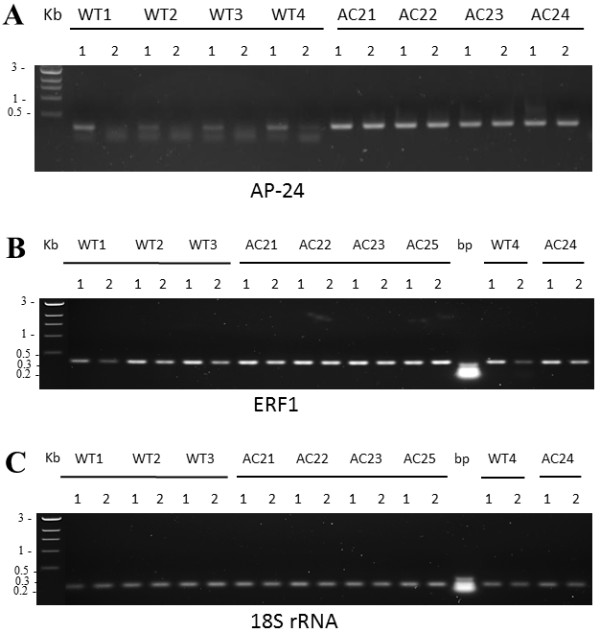
**Detection of methylation using methylation sensitive restriction enzyme cutting combined with PCR amplification.** Chromosomal DNA of wild type and of AC2 expressing tobacco leaves were isolated and cut with either *Bam*HI (1) or *BamHI* and *Hpa*II (2) restriction enzymes. DNA fragments were amplified using primers presented in Additional file 11. Osmotin, AP-24 (**A**) and ethylene responsive factor1 ERF1 (**B**) indicated methylation in AC2 DNA fragments, whereas no methylation was detected in 18S rRNA (**C**) AC2 DNA fragments compared to wild type DNA samples. Data consists of four biological replicates.

To investigate possible alterations in the level of DNA methylation level, we also performed PCR-amplification experiments over some genomic DNA regions that contained at least three methylation sensitive restriction enzyme sites. These analyses indicated increased methylation at least in the coding regions of ERF1 and AP24 genes but not in 18S RNA (Figure 8). However, other gene fragments showed no change in the methylation status (data not shown).

Neither genes encoding S-adenosyl-L-methionine (SAM) biosynthesis nor genes encoding SAM-recycling were down regulated as much as in HC-Pro expressing plants [26]. Thus, it appears that recycling of SAM-provided methyl groups for trans-methylation reactions was not affected in AC2-expressing plants. Interestingly, a large group of histone transcripts were down-regulated in AC2 expressing tobacco plants (Table 6, Additional file 12), while a similar group of histone transcripts were up-regulated in HC-Pro expressing tobacco plants. Also some histone acetyl-transferases were up-regulated, possibly leading to opening of the chromatin structures and thus increasing transcriptional activity. Another interesting phenomenon was the down-regulation of cell cycle-related transcripts in AC2 expressing plants, as similar transcripts were up-regulated also in the HC-Pro expressing plants (Additional file 12).

## Discussion

In this work we have analysed the transcriptome profile of transgenic tobacco plants expressing the AC2 RSS derived from ACMV, nowadays classified as a member of the genus Begomovirus, and shown that this single RSS protein causes massive changes in the gene expression. These changes may have been caused trough at least three identified regulatory nodes, which all interfere with large metabolic networks. The first node is the induction of plant hormones like jasmonates and ethylene, leading to jasmonate and ethylene responsive gene expression. Both jasmonates and ethylene are known to be highly involved in defence and stress regulated gene expression, and jasmonate hormones are also responsible for inducing oxidative stress [30,35]. The second regulatory node appears to be related to photosynthetic end products leading to changes in the transcriptional and translational regulation. The role of chloroplasts in this retrograde signalling is crucial. Results demonstrated that photosynthetic light and dark reactions were clearly down-regulated. The third regulatory node appears to consist of silencing directed modifications in cell cycle including changes in transcripts involved in DNA replication and chromatin structures.

### Defence and stress reactions in transgenic AC2 expressing tobacco plants

The expression of the AC2 RSS in transgenic tobacco plants induced more pronounced defence and stress related gene expression than was found in plants expressing HC-Pro RSS in our previous study [26]. Although many similar defence and stress related transcripts were induced in these two types of transgenic plants, a high number of transcripts were enhanced only in the AC2 expressing plants, the most prominent difference being the induction of transcripts related to scavenging of ROS in these plants (Table 3, Additional file 8). These included transcripts coding for scavengers of hydrogen peroxide (H_2_O_2_) and of superoxide related oxygen radicals. The presence of ROS also was indicated by spontaneous HR lesions in the young leaves (Figure 3). Also, transcripts related to systemic acquired resistance (SAR) were induced, maybe via induction of avirulance (Avr) determinant related genes (Table 3) (reviewed in [36]). Balance in the redox homeostasis seems also be affected in AC2 RSS expressing transgenic tobacco plants, as several genes that function in balancing the redox state in the cells, like glutathione-S-transferases, glutaredoxins and thioredoxins were up-regulated in the leaves (Table 3). All in all, this may have affected the redox-responsive gene regulation in the AC2 RSS expressing tobacco cells [37].

### Photosynthesis is also regulated by jasmonates

Photosynthesis is the key regulator of almost all metabolic and differentiation responses in the plant cells but it has also a major role in regulating gene expression by retrograde signalling. Our results clearly pointed out that many in photosynthesis related transcript (Table 4), and the photosynthetic oxygen evolution (Figure 4) were significantly decreased in AC2 expressing plants. Interestingly, jasmonate hormones have been shown to regulate photosynthesis related transcripts in *Arabidopsis*[38], to down-regulate the oxygen evolution (i.e. the Hill reaction activity) in isolated barley thylakoids [39], to up-regulate defence, stress, senescence and cell wall related transcripts and to causes oxidative stress in plants and thus also to change redox state of proteins [37]. Thus all these observed effects in AC2 expressing plants could have been related to up-regulated jasmonate biosynthesis [38]. Further on, the reduced photosynthesis may have changed the sugar metabolism and thus caused major secondary effects on retrograde signalling between the nucleus and the chloroplast. For instance, it may explain the changes in protein synthesis (e.g. ribosomal proteins, translation factors etc.), cell wall synthesis, and chromosome/histone modifications related transcripts (Tables 5 and 6, Additional file 7) in the AC2 expressing plants.

Several studies have shown that energy deprivation and different stresses change energy-dependent transcriptional regulation [40,41] with drastic down-regulation of transcripts related to different biosynthetic processes and especially protein syntheses (e.g. translation factor and ribosomal protein encoding transcripts) and up-regulation of catabolic reactions to restore energy deprivation on the cells.

### Protein synthesis affected in AC2 expressing plants

Results clearly indicated that expression of AC2 protein in transgenic plants had a major effect on transcripts related both protein synthesis and protein degradation (Figure 7). The down-regulation of protein synthesis caused to some extend by reduction of levels of multiple transcripts, but also the down-regulation of genes encoding both nuclear and plastid ribosomal proteins, ribosomal RNA or translation factors (Tables 4,5 and Additional file 7 and Additional file 12). Not only synthesis of proteins but also degradation of proteins was affected. Over and under presentation analysis (Additional file 7) indicated that both cysteine and aspartate proteases were up-regulated in leaves of AC2 RSS expressing plants. There were also indications of proteasomal protein degradation (Additional file 12). Interestingly, it has suggested that the geminiviral C2 protein might be the key player in proteasomal regulation of protein degradation [42,43]. It was shown that C2 protein is capable of binding to proteins involved in proteasomal complexes e.g. SCF E3 ligase complexes and thus inhibiting jasmonate related gene expression in *Arabidopsis* (See also [44]). However, our results clearly indicate that the geminiviral protein AC2 has an opposite effect; enhancement of jasmonate signalling and jasmonate responsive gene regulation [45] and no effects to transcripts related to neither SA synthesis nor SA responsive gene expression [46].

### Cell cycle, genome methylation and histone expression is altered in AC2 expressing transgenic plants

Begomoviruses are known to replicate their single-stranded DNA genomes through double-stranded DNA intermediates that are associated with cellular histone proteins to form minichromosomes [24]. Thus it is important to Begomoviruses to induce the accumulation of the DNA replication machinery in mature plant cells. This is achieved most likely by modifying cell cycle and transcriptional regulation, for instance, the infection of *cabbage leaf curl geminivirus* (CaLCUV) has been shown to induce changes in the cell cycle in virus infected cells [47]. Interestingly, our results also suggested that expression of AC2 RSS may have an effect on cell cycle (Additional file 12), providing replication-competent environment and preventing expression of genes needed for mitosis [48,49].Our results demonstrated that the expression AC2 RRS in transgenic plants up-regulated transcripts involved in DNA methylation (KTF1 and AGO5) and down-regulated transcripts involved in histone synthesis (Table 6). This suggested that, although the transcriptional activator AC2 suppresses silencing (DNA methylation) in the native host(s) of ACMV, and apparently enhances the virus replication in these hosts, in tobacco (non-host to ACMV) it induces defence mechanisms, leading to opposite direction, i.e. to increase of methylation and decrease of the histone synthesis.

## Conclusions

The expression of AC2 RSS in transgenic tobacco plants induced clear phenotypic changes in tobacco plants, even though tobacco is not considered as native host for ACMV. This study indicated several possible action mechanisms of the AC2 RSS. First both jasmonate and ethylene hormone responsive regulatory cascades were induced, secondly, the rate of photosynthesis and also the carbon metabolism were reduced. The stress and defence responsive gene expression was enhanced including both induction and response of reactive oxygen species (ROS). All these regulatory nodes may have been interconnected to one another. Also, changes occurred in regulation of cell cycle and transcription as well as changes in the genomic architecture like DNA methylation and abundance of histones. Thus it seems that the silencing suppression activity of AC2 in tobacco is indicated in the enhancement of the jasmonate and ethylene biosynthesis, known to be regulated and repressed via RNA silencing [50]. Up-regulation of these plant hormones was related to induction of a large variety of stress-related genes, and severe developmental defects in the plants. Interestingly, the natural silencing suppression mechanism of the geminiviral RSS, i.e. reduction of the DNA methylation, did not occur in this non-host plant, but rather the opposite effect was observed, apparently related to the induced plant defences.

## Methods

### Plant material

The wild type tobacco (*Nicotiana tabacum*), empty vector tobacco control (pBIN61), and transgenic tobacco plants expressing AC2 transgene [27]were grown in greenhouse conditions at 60% relative humidity and 22°C, with a day/night regime of 16h light (150 μmol photons m^-2^s^-1^) and 8 h dark. Leaf samples (third leaf from the top) were taken from one-month-old plants, the plants were at that time about 20 centimetres of height. Leaf and flower samples were taken from the same plant. Flower samples were taken one day prior to opening. Both leaf and flower samples were directly frozen in liquid nitrogen and stored at −80°C.

### RNA extraction, cDNA labelling, microarray hybridization and scanning of the microarray chips

Total RNA was isolated from leaves and flowers of wild type and transgenic plants using TRIsure-reagent (Bioline, UK) according to manufacturer’s recommendations. Total RNA was further purified using RNeasy clean up column (QIAGEN inc. USA). The cDNA labelling, the quality checking of total RNA and labelled cDNAs, hybridization on a Agilent’s 4x44K tobacco chip (Design ID 21113), washing and the scanning the chips was performed in a similar fashion, concurrently with already published HC-Pro microarray samples [26].

### Microarray data analysis

All data handling was performed using Chipster, a visual program based on R Project for Statistical Computing program (Center of Scientific Calculating (CSC), Finland) ( [51], Agi4x44k preprocess, Lopez-Romero, 2010). In order to compare intensity values of different samples, both wild type and empty vector control tobacco samples (6) vs. transgenic AC2 expressing leaf samples, (3), were normalized together. The flower samples were normalized together in a similar fashion. Normalization of three biological replicates was performed using median signal values and median background values. A background offset value (50) was added to prevent negative values during normalization. Normalization of the arrays was performed using a “quantile” parameter. The array results have been deposited into ArrayExpress with accession number E-MEXP-3724. Differentially expressed transcripts were re-annotated as was performed in earlier publication by Soitamo et al. 2011 [26]. Microarray results were visualized by using MapMan program and enrichment analysis of functional categories by using PageMan program [52,53].

### Verification of differentially expressed genes

The array results were verified by using RT-qPCR according to MIQE guidelines [28]. The RT-qPCR was performed from the same RNA samples as were previously used in microarray experiments. The cDNA was synthesized from 1 μg of purified leaf or flower total RNA using RevertAid H-Minus M-MuLV reverse transcriptase according to manufacturer’s recommendations (Product # EPO451, Fermentas). Produced cDNA was diluted and 3μl was used in RT-qPCR (Maxima SYBR Green /Fluorescein qPCR MasterMix (2X) (Product # KO242, Fermentas). The gene specific reference and sample primers used in RT-qPCR are listed in Additional file 11. For each three biological replicates, three-four technical replicates were run to minimize pipetting errors. RT-qPCR reactions were run in a 96-well plate containing both wild type (reference) and AC2 transgenic samples. The RT-qPCR was performed using Bio-RAD’s iQ5 machine. The results were calculated using the quantification cycle (Cq) method (delta delta Cq) according to Bio-RAD’s iQ5 default settings. All primer pairs produced only one peak in DNA melting curves indicating high specificity of primers. Standard error of mean (s.e) was also calculated of three to five biological replicates.

### Detection of oxygen radicals in leaves

Hydrogen peroxide and superoxide anions were detected by using stains that react with these radicals. DAB (0.1mg/ml DAB pH to 3.8 using NAOH) was used to detect presence of hydrogen peroxide and NBT (0.1mg/ml NBT in 25mM HEPES/KOH pH 7.4) was used to detect presence of superoxide radicals [54]. Staining whole leaves was performed in 20ml DAB or NBT solution in a small Petri dish. Leaf was cut from the stalk under staining solution to get a new surface area for stain infiltration. Leaves were then kept in the dark overnight. Next morning, chlorophyll was removed from the leaf using 96% ethanol. Ethanol was changed a few times, until all chlorophyll was removed (took at least 24h). Pictures of leaves were taken from which all chlorophyll was removed.

### Photosynthetic and chlorophyll quantification measurements

Equal amount of intact wild type and HC-Pro transgenic tobacco leaves (1.0 g) were ground in an ice cold mortel in 4 ml of thylakoid isolation buffer (0.3 M sorbitol, 50 mM Hepes/KOH pH 7.4, 5 mM MgCl_2_, 1 mM EDTA and 1% BSA). Suspension was filtered through a Miracloth and 2 ml thylakoid suspension was pelleted in Eppendorf-centrifuge 12 000xg for 2 minutes. The pellet was resuspended into 100 μl of O_2_-electrode measuring buffer (0.3 M sorbitol, 50 mM Hepes/KOH pH 7.4, 5 mM MgCl_2_, 1 mM KH_2_PO_4_). Oxygen evolution was measured directly in a Clark type O_2_-electrode using 0.5 mM DCBQ as electron donor. The chlorophyll concentration was calculated according to Porra et al. [55]. Samples in the cuvette were quantified based on equal amount of total chlorophyll. The anthocyanin concentration was measured from three 8 mm leaf discs of wild type and of AC2 expressing tobacco plants according to Neff and Chory [56].

### Isolation of proteins and 2D-PAGE

Protein samples of leaves from wild type and HC-Pro expressing transgenic plants were isolated concurrently with the RNA isolation using TRIsure-reagent (Bioline). The protocol was adapted from TRIzol (Invitrogen inc. USA) and performed according to manufacturer’s recommendations. The protein concentration was measured using Lowry method. Proteins were first separated by Bio-Rad laboratories 7cm IPG strips pH 3–10 according to manufacturer’s recommendations. 250 μg of protein was loaded per a strip. Strips containing wild type and transgenic HC-Pro focused protein samples were then run simultaneously in a large gel in Protean II apparatus (Bio-Rad) to produce a similar mobility of focused proteins of both strips. Protein gels were then fixed and stained in colloidal Coomassie blue stain (PageBlue staining kit, Fermentas) according to manufacturer’s recommendations, destained and photographed.

### Detection of DNA methylation using PCR amplification after DNA methylation sensitive restriction enzyme cutting

DNA was isolated from the leaves of wild type and AC2 expressing tobacco leaves according to protocol of Genomic DNA from Plant (Nucleospin Plant II, Macherye-Nagel Germany). DNA was cut with either *Bam*HI or *Bam*HI and *Hpa*II restriction enzymes. The cut fragments were amplified using *Taq* polymerase with 2 mM MgCl_2_. Primers for amplification are given in Additional file 11. Primers were designed so that the fragments contained three CCGG sites inside amplified region. Methylation in the *Hpa*II sites in the DNA fragments prevented the cleavage of the DNA and an amplified PCR product was detected.

## Competing interests

The authors have no non-financial, financial or patent related competing interests.

## Authors’ contributions

AJS grew and collected the plant material for the experiments. The work was planned by KL. The experimental work and the reannotation of significantly altered transcripts were carried out by AJS and BJ together. AJS and KL wrote the manuscript. All authors have read and approved the manuscript.

## Supplementary Material

Additional file 1 The expression of AC2 transcripts in transgenic plants is measured by using RT-qPCR. Click here for file

Additional file 2 ** Quality control of intensity values in leaf and flower samples.** Graphical Box Plot presentation of microarray data of intensity values originating from leaf and flower samples of wild type and transgenic AC2 expressing plants. Click here for file

Additional file 3 Up-regulated transcripts of leaf samples in transgenic AC2 expressing plants. Click here for file

Additional file 4 Down-regulated transcripts of leaf samples in transgenic AC2 expressing plants. Click here for file

Additional file 5 Up-regulated transcripts of flower in transgenic AC2 expressing plants. Click here for file

Additional file 6 Down-regulated transcripts of flower in transgenic AC2 expressing plants. Click here for file

Additional file 7 ** Over and under presentation analysis of functional categorization of leaves expressing AC2 or HC-Pro RSS.** Analysis was performed using PageMan program (MapMan 3.5.1R2). Numbers after functional categorization indicate log2 values of differentially expressed genes and intensity of coloured boxes the z-scores of p values with FDR<0.05 [53]. Click here for file

Additional file 8 ** Visual presentation of transcripts involved in biotic stress.** Data consists of up or down regulated transcripts with p-values less than 0.05 (FDR) in leaf and flower samples expressing AC2 or HC-Pro RSS. Click here for file

Additional file 9 The amount of chlorophyll and anthocyanin in leaves of wild type and in AC2 expressing plants. Click here for file

Additional file 10 The amount of total protein against fresh weight (FW) and dry weight (DW) in wild type and AC2 expressing tobacco leaves. Click here for file

Additional file 11 PCR primers for RT-qPCR and methylation sensitive restriction enzyme amplified PCR. Click here for file

Additional file 12 ** Visual presentation of transcripts involved in cellular responses overview.** Data consists of up or down regulated transcripts in leaf samples expressing AC2 or HC-Pro RSS**.** Click here for file
