# Supplementary material for: Expression of geminiviral AC2 RNA silencing suppressor changes sugar and jasmonate responsive gene expression in transgenic tobacco plants
Source: BMC Plant Biol. 2012 Nov 7;12:204. doi: 10.1186/1471-2229-12-204 (PMC3519546; doi:10.1186/1471-2229-12-204)

LEAF ALL TRANSCRIPTS

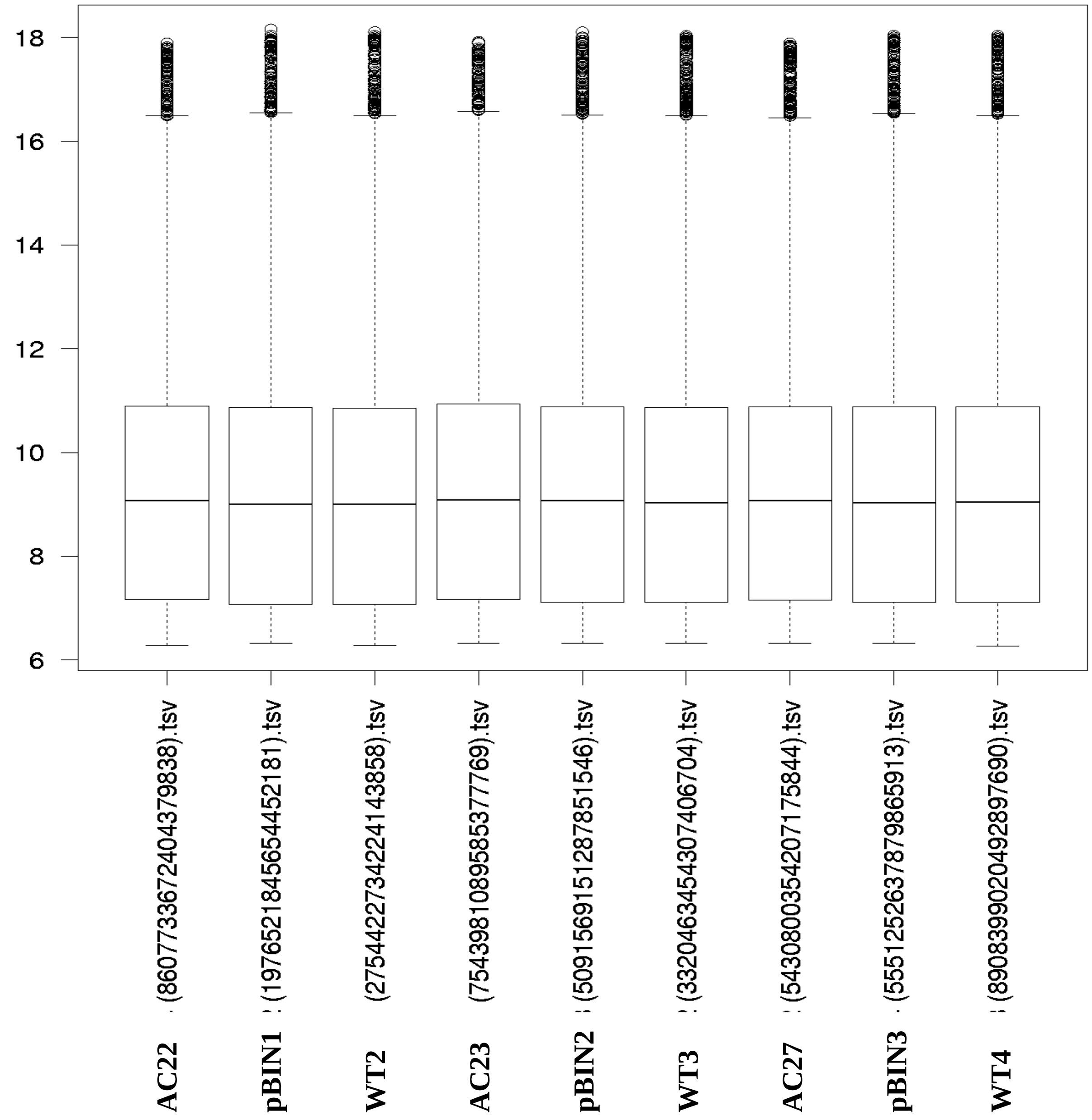

LEAF UP-REGULATED TRANSCRIPTS

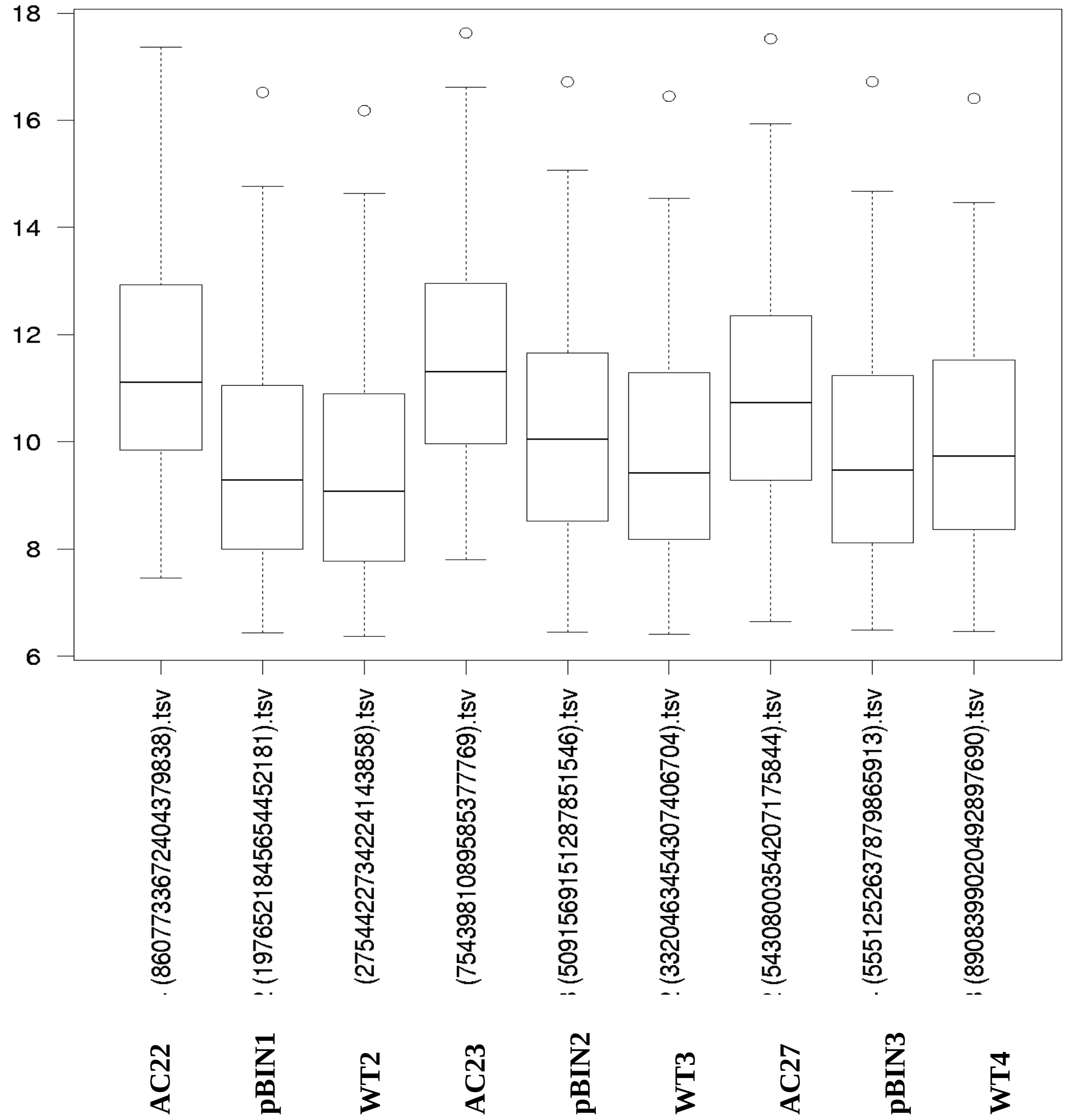

LEAF DOWN-REGULATED TRANSCRIPTS

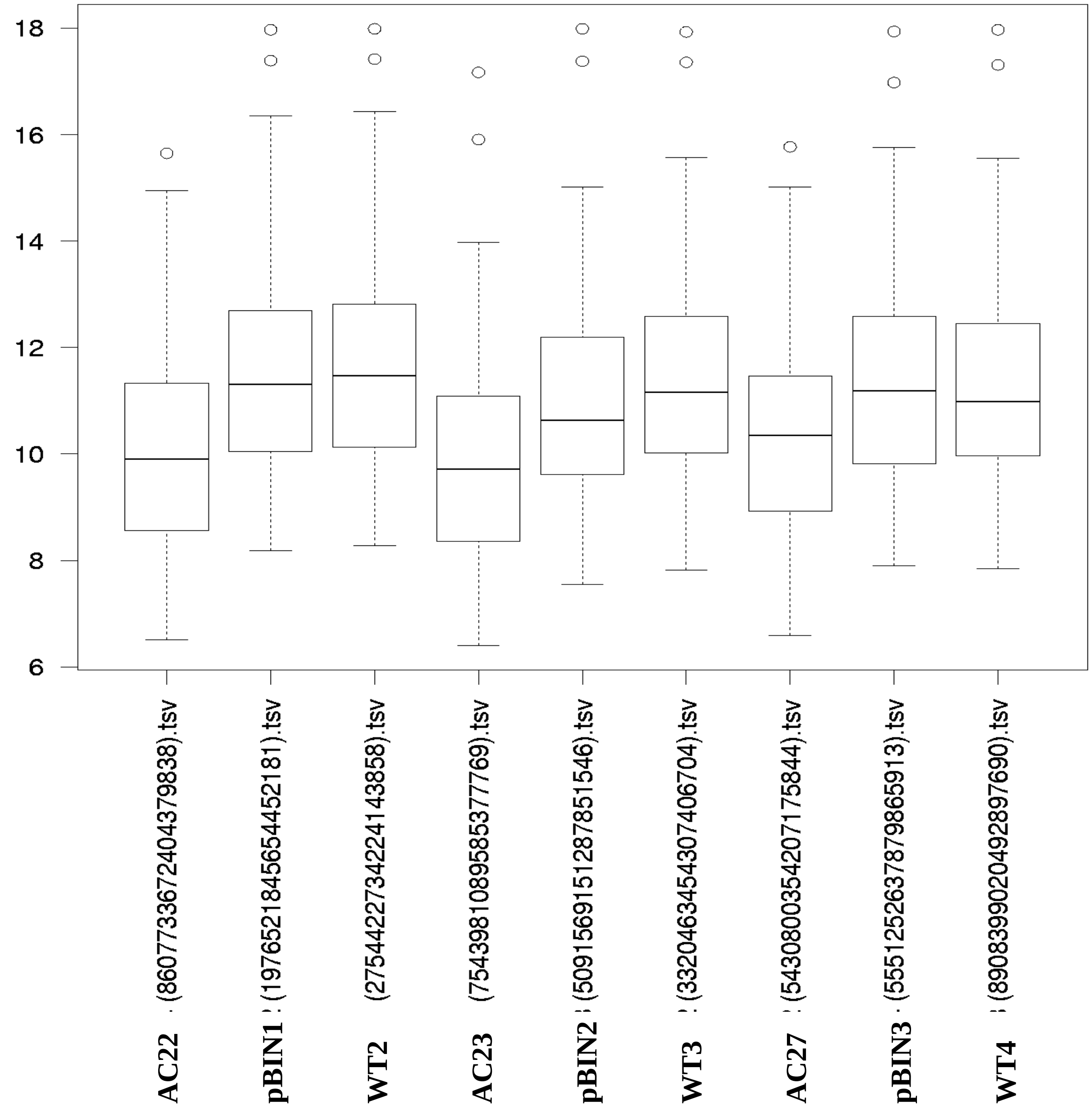

FLOWER ALL TRANSCRIPTS

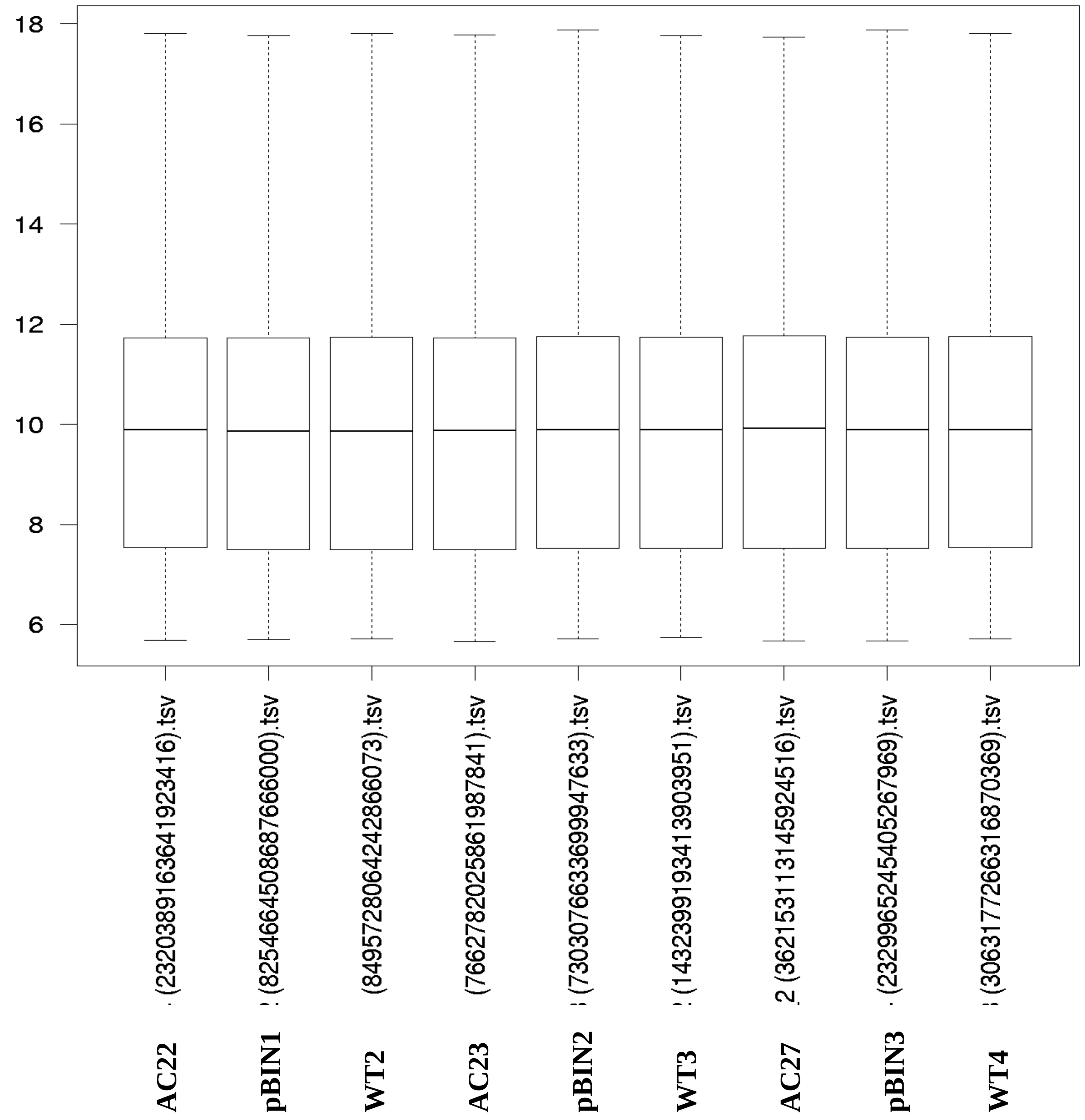

FLOWER UP-REGULATED TRANSCRIPTS

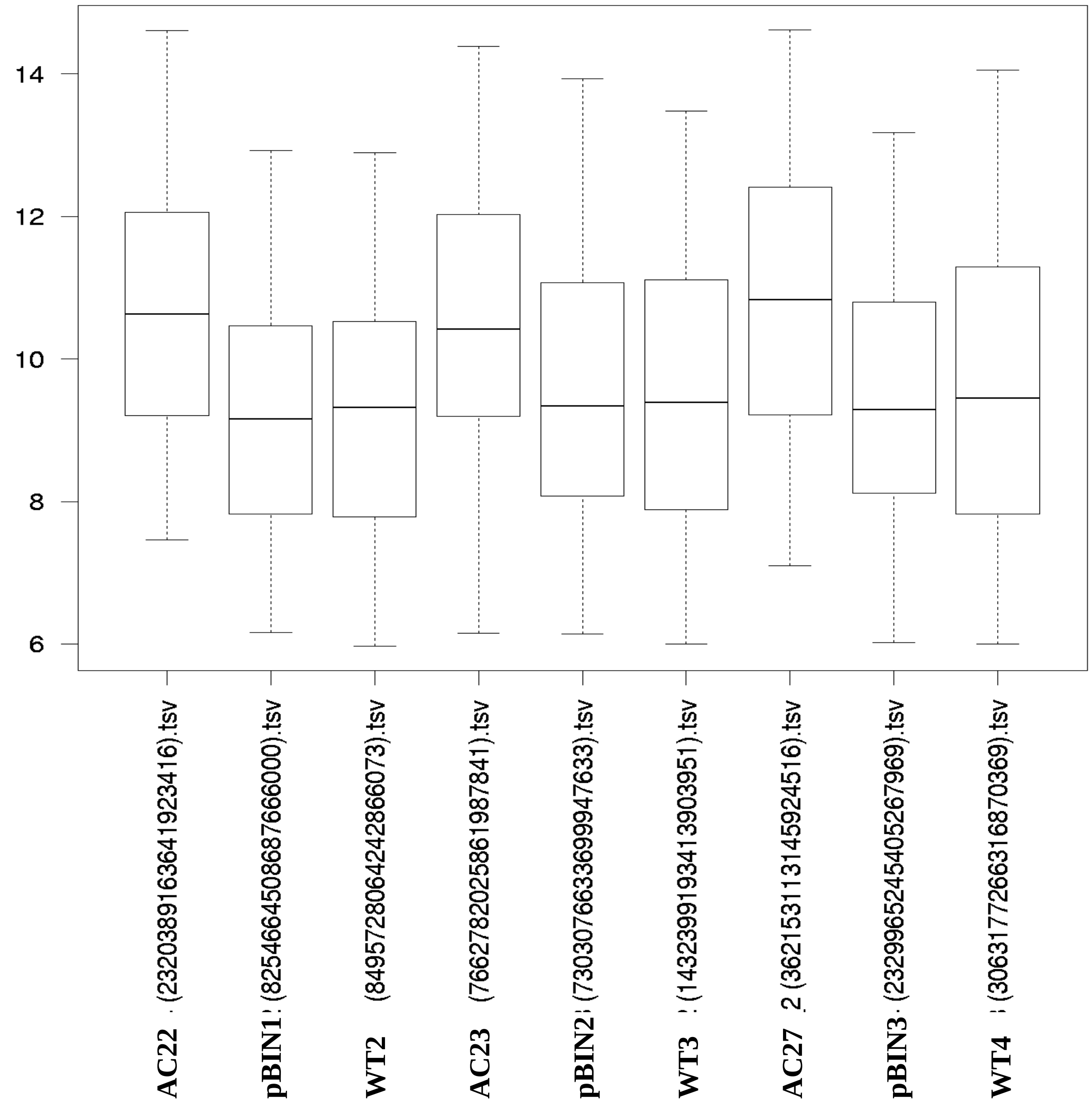

FLOWER DOWN-REGULATED TRANSCRIPTS

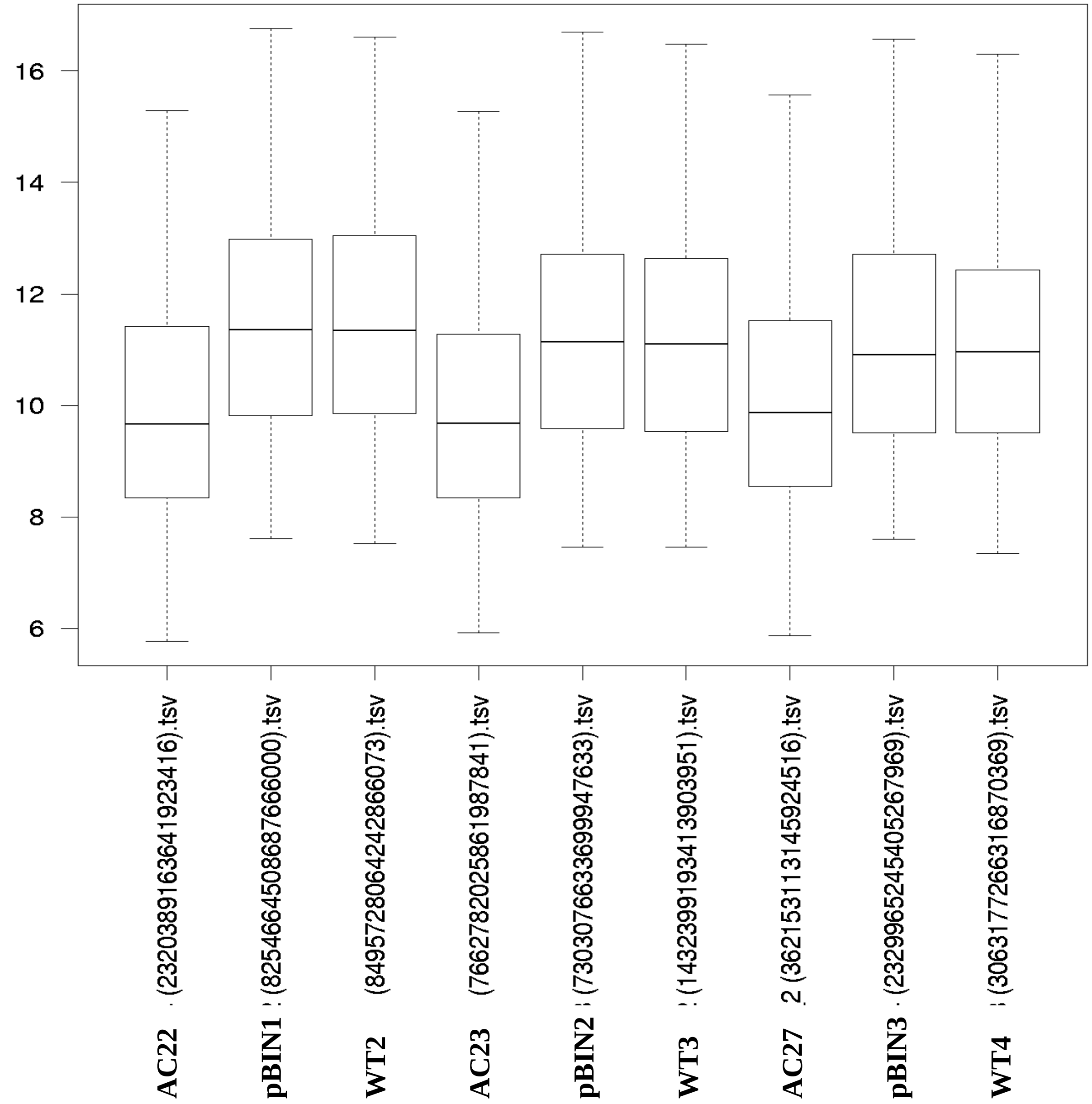

Supplement: Additional file 2 — Quality control of intensity values in leaf and flower samples. Graphical Box Plot presentation of microarray data of intensity values originating from leaf and flower samples of wild type and transgenic AC2 expressing plants. [file 1471-2229-12-204-S2.pdf]
