# Supplementary figures and images for: Expression of geminiviral AC2 RNA silencing suppressor changes sugar and jasmonate responsive gene expression in transgenic tobacco plants
Source: BMC Plant Biol. 2012 Nov 7;12:204. doi: 10.1186/1471-2229-12-204 (PMC3519546; doi:10.1186/1471-2229-12-204)

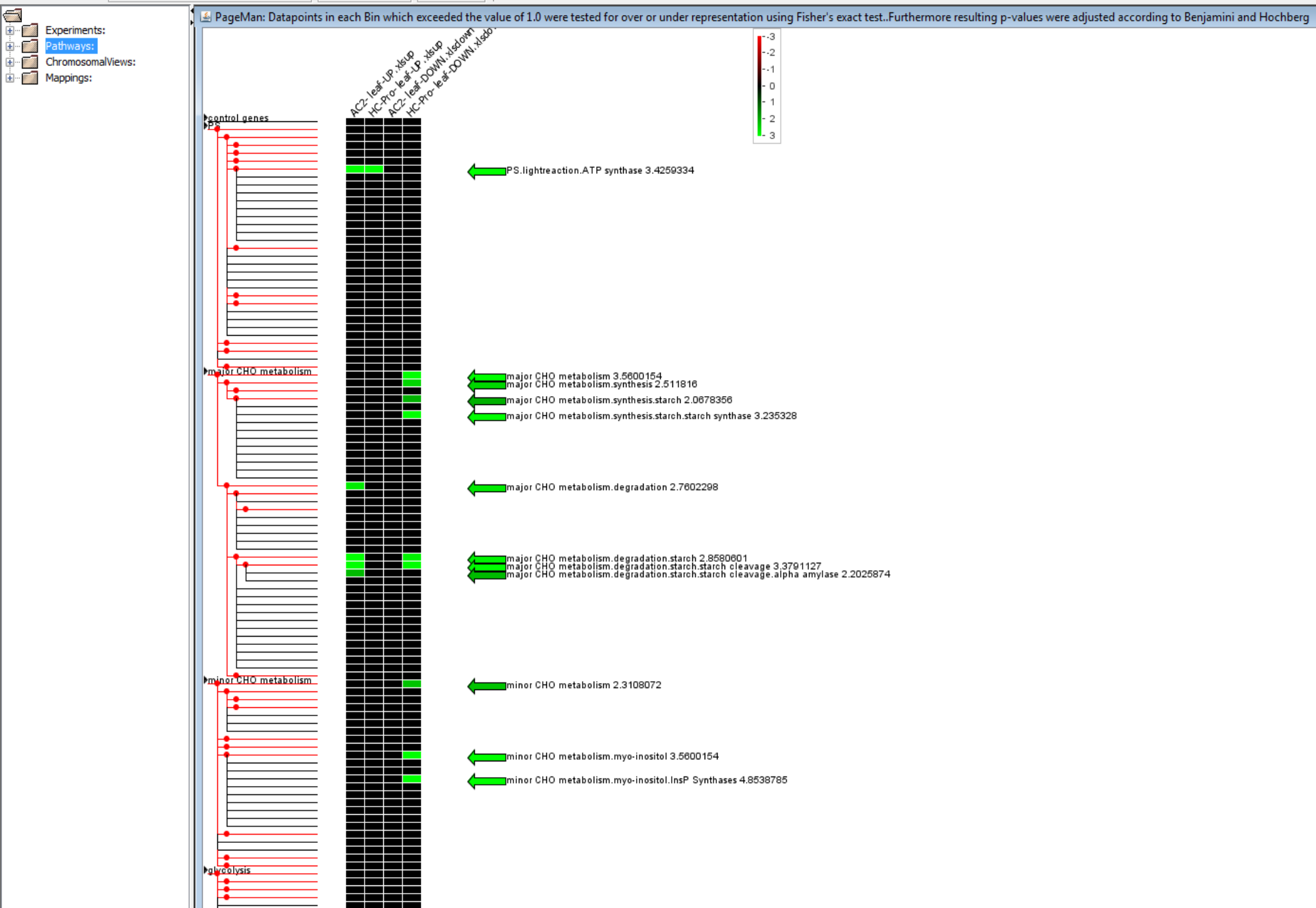

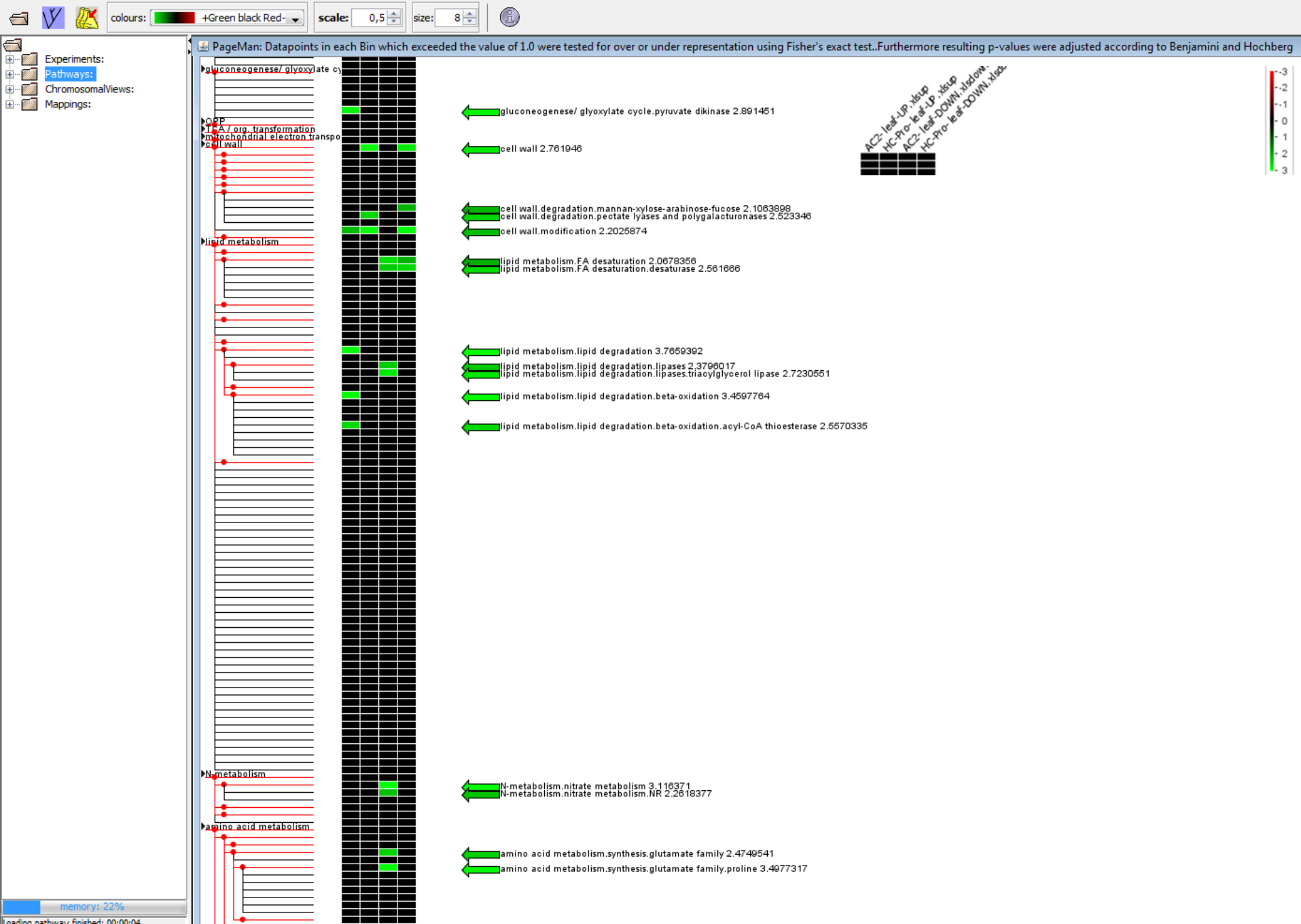

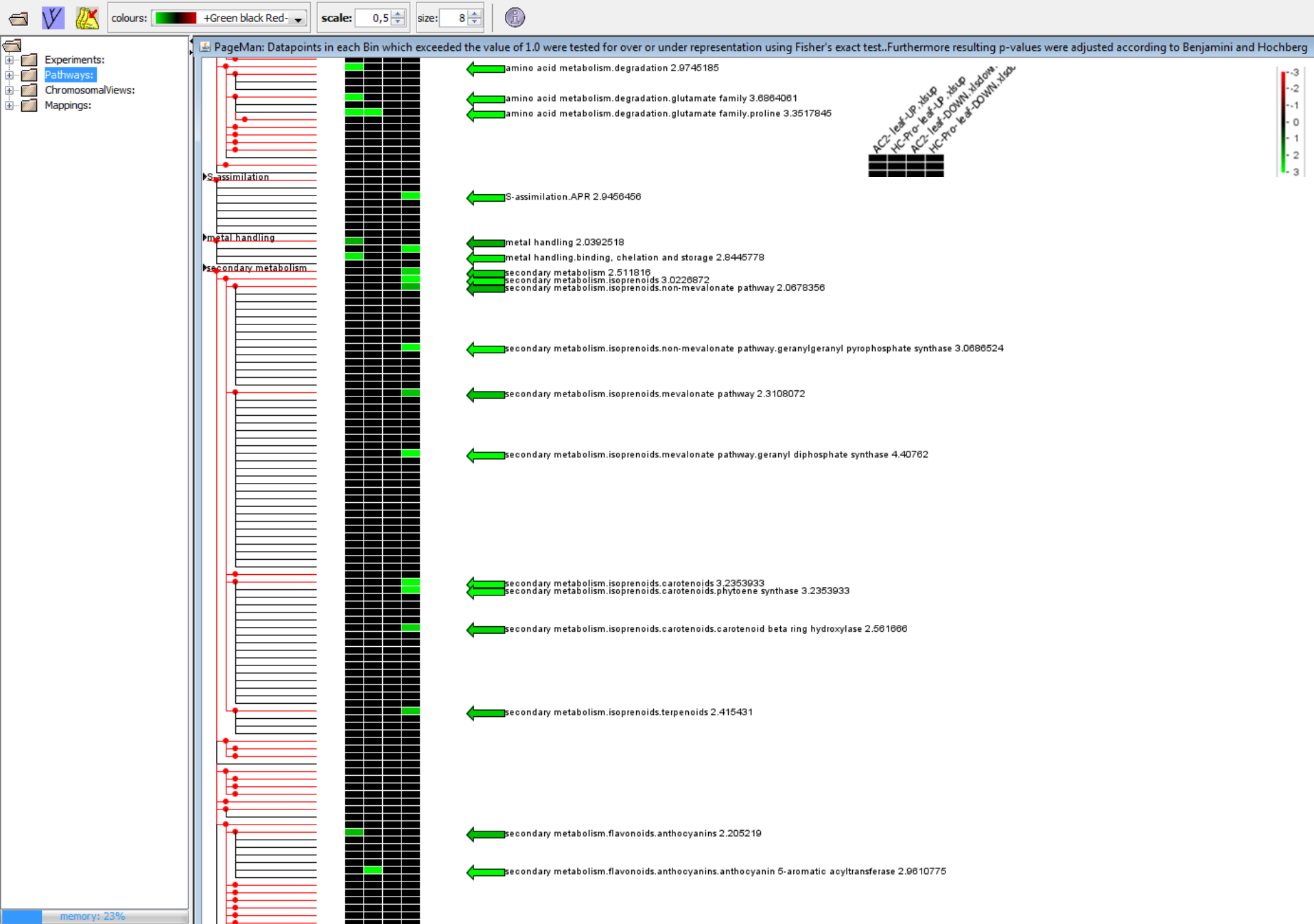

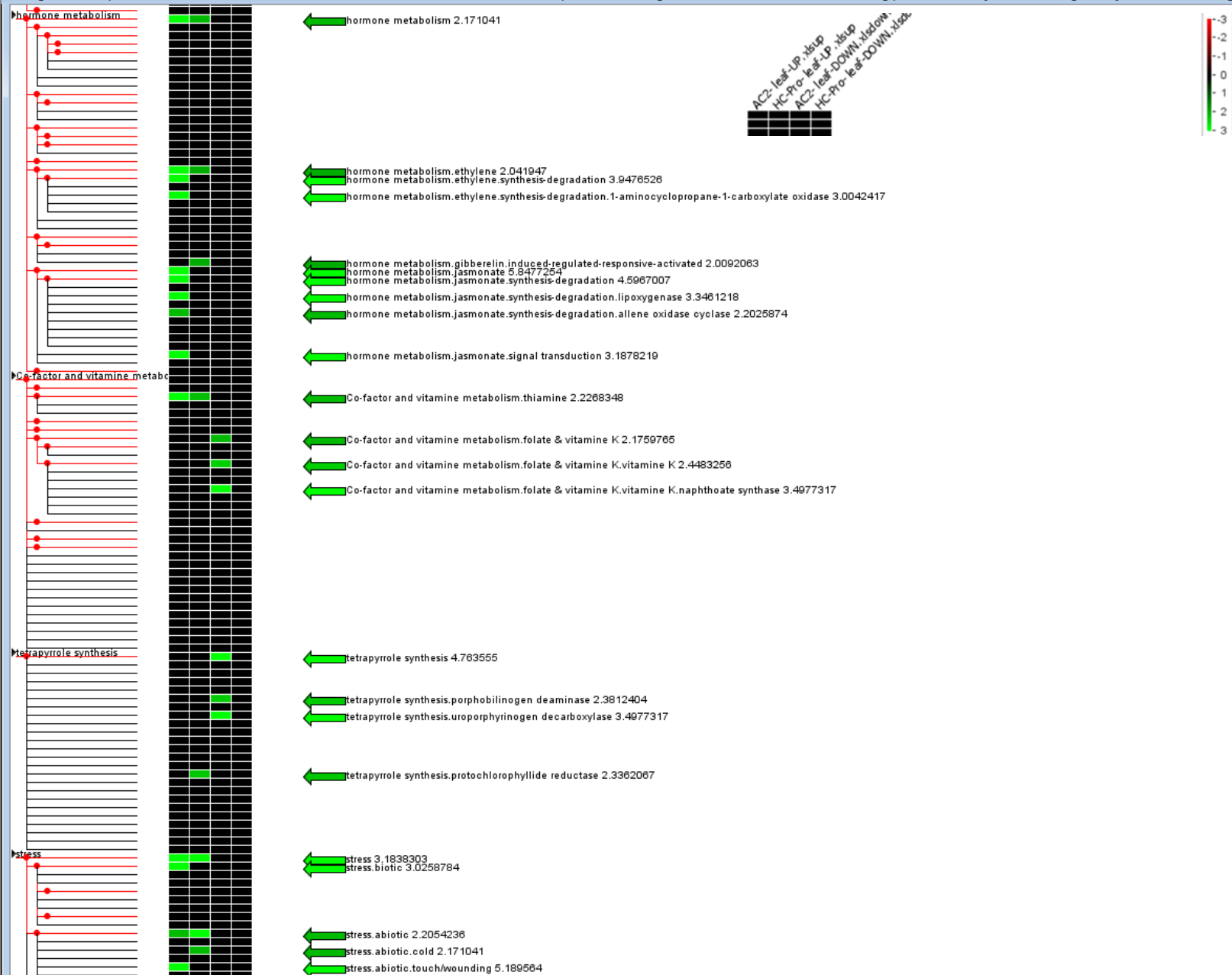

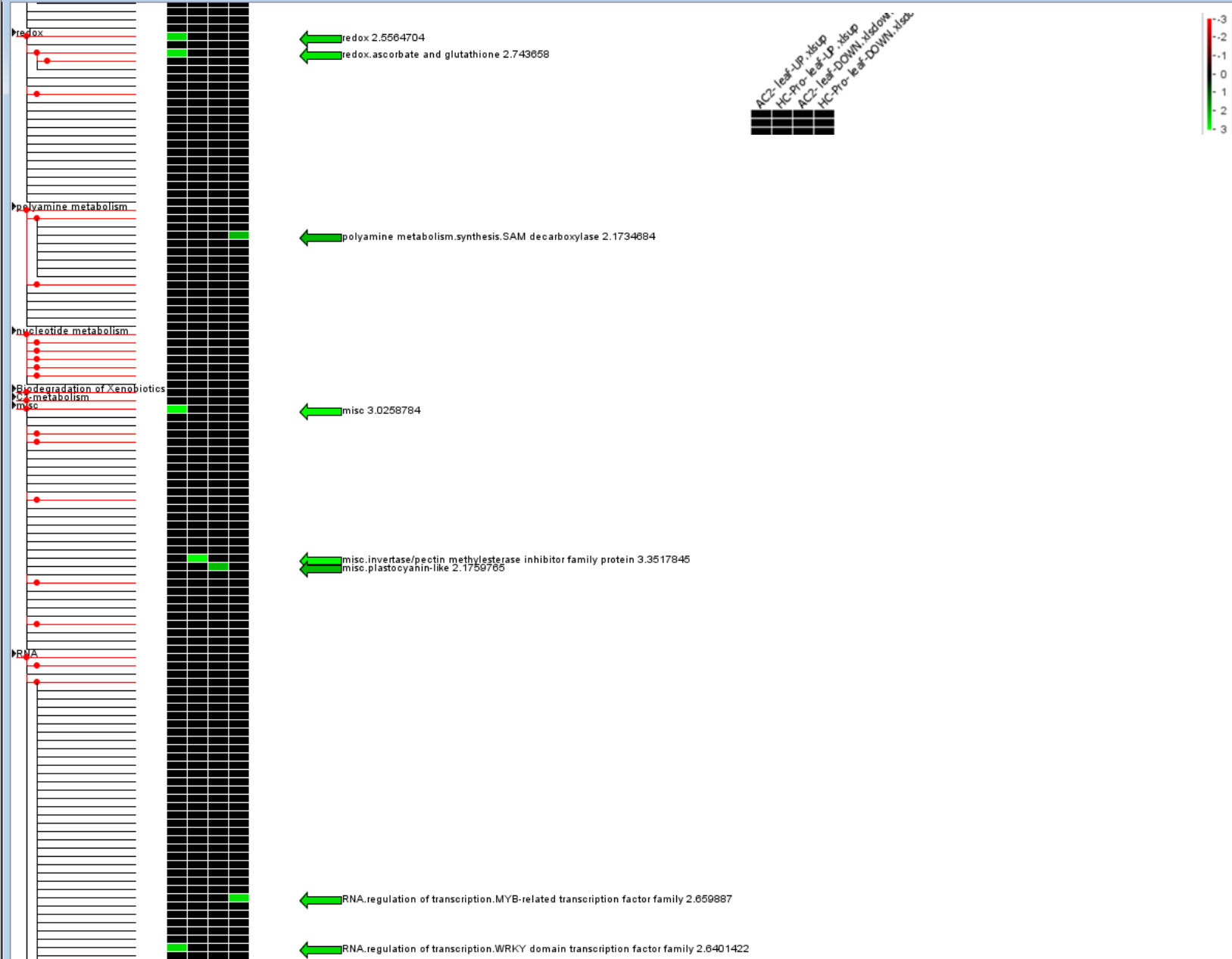

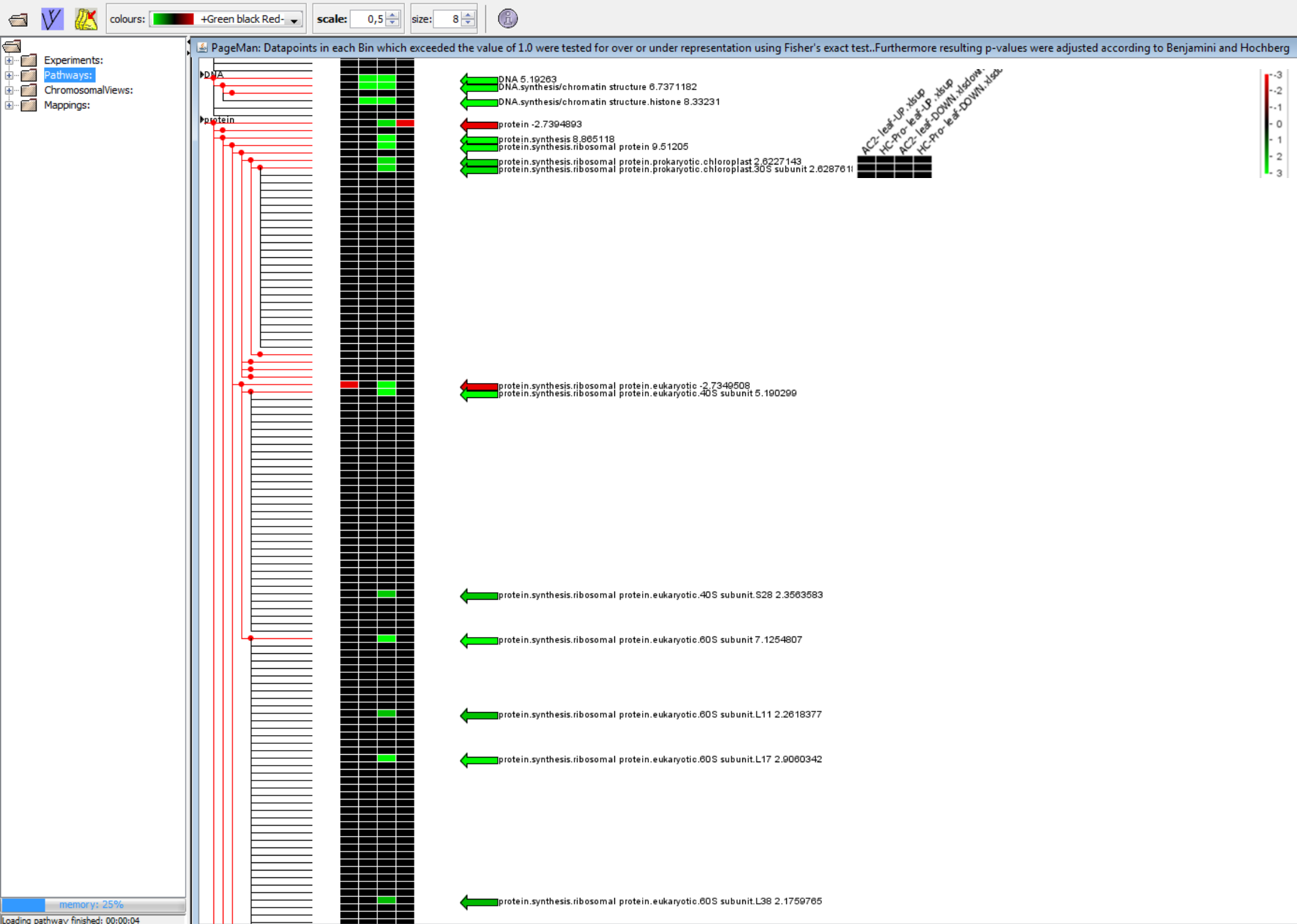

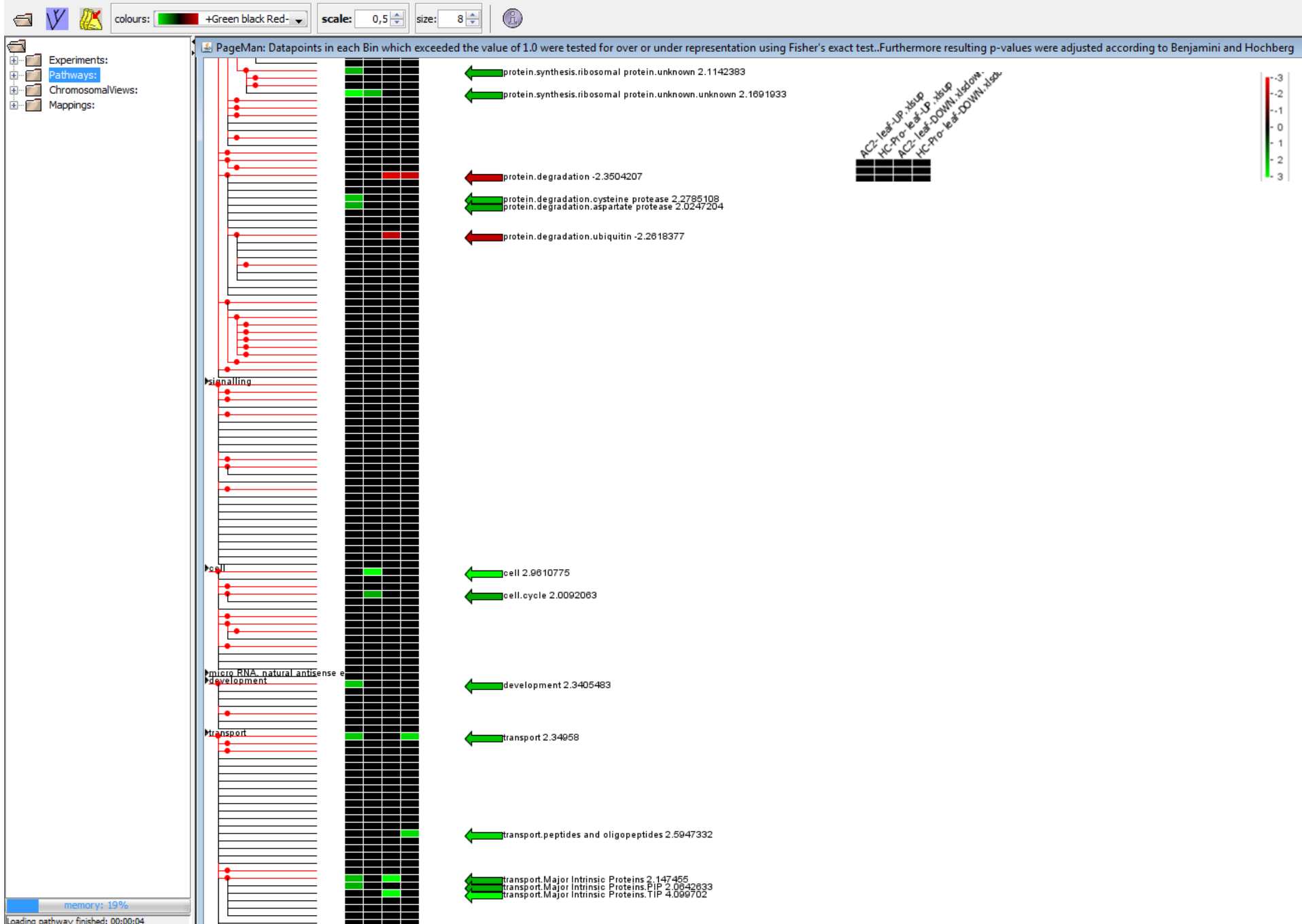

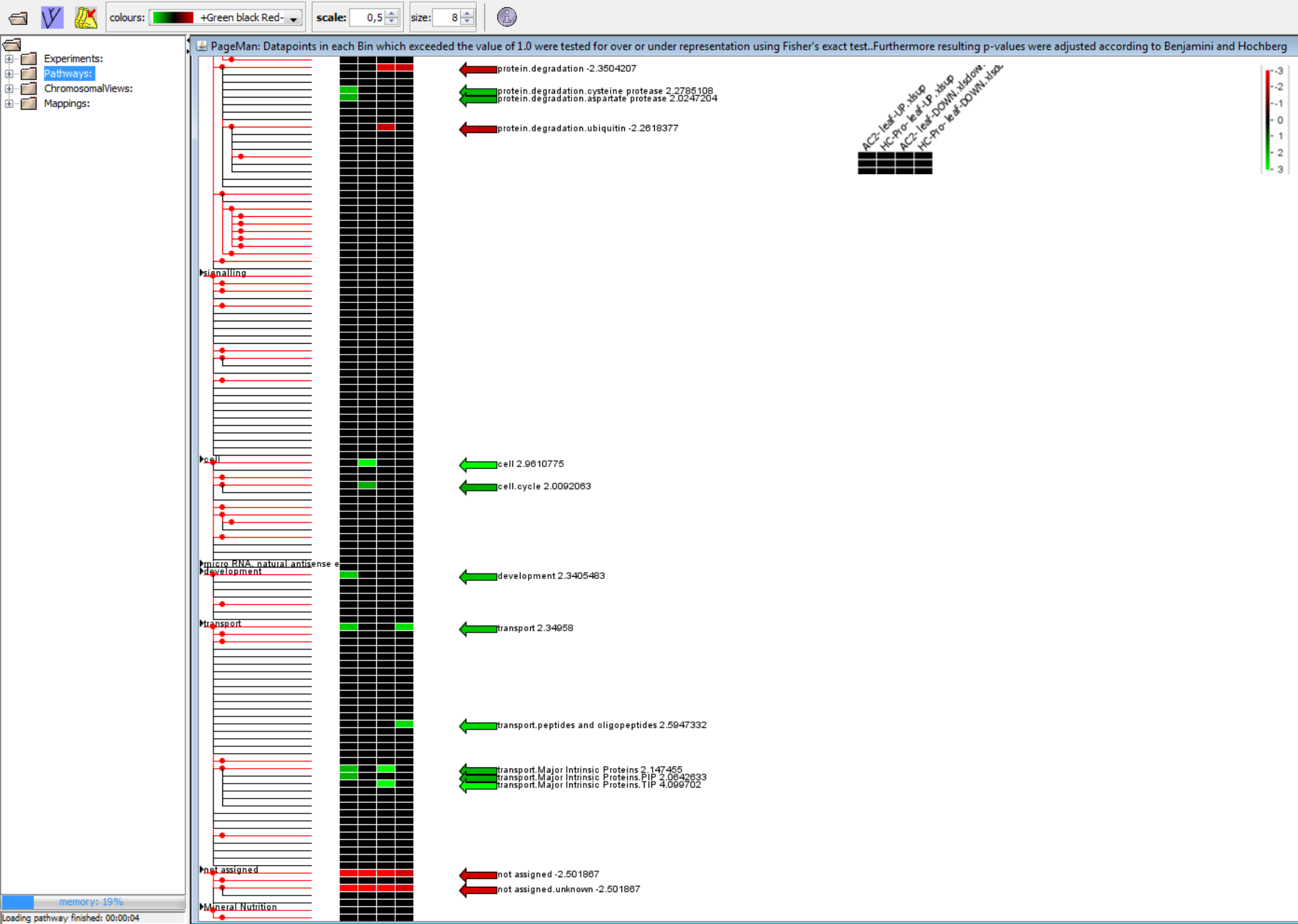

Supplement: Additional file 7 — Over and under presentation analysis of functional categorization of leaves expressing AC2 or HC-Pro RSS. Analysis was performed using PageMan program (MapMan 3.5.1R2). Numbers after functional categorization indicate log2 values of differentially expressed genes and intensity of coloured boxes the z-scores of p values with FDR<0.05 [53]. [file 1471-2229-12-204-S7.pdf]
