# Supplementary material for: Expression of geminiviral AC2 RNA silencing suppressor changes sugar and jasmonate responsive gene expression in transgenic tobacco plants
Source: BMC Plant Biol. 2012 Nov 7;12:204. doi: 10.1186/1471-2229-12-204 (PMC3519546; doi:10.1186/1471-2229-12-204)

Biotic stress- AC2 leaf

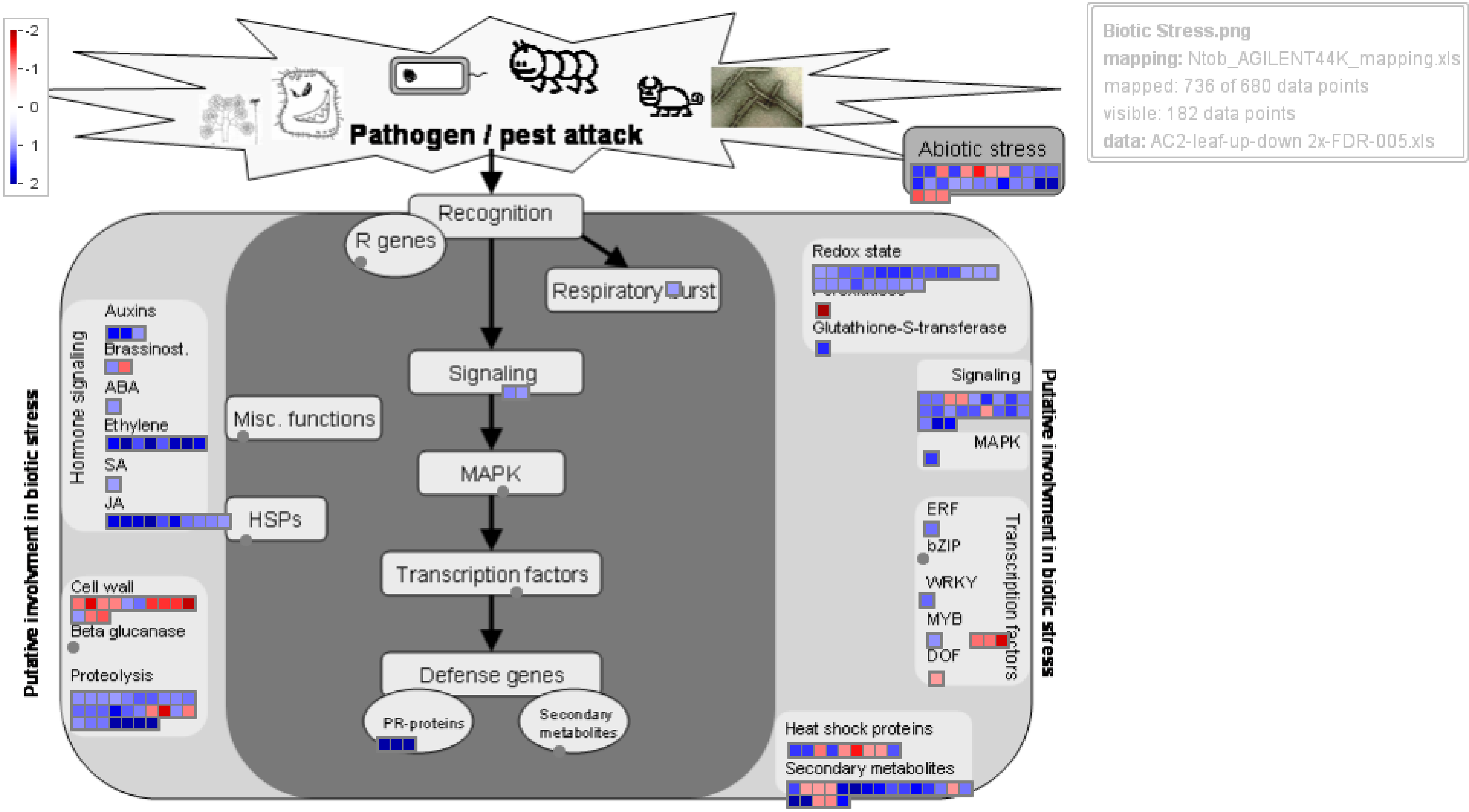

Biotic stress- AC2 flower

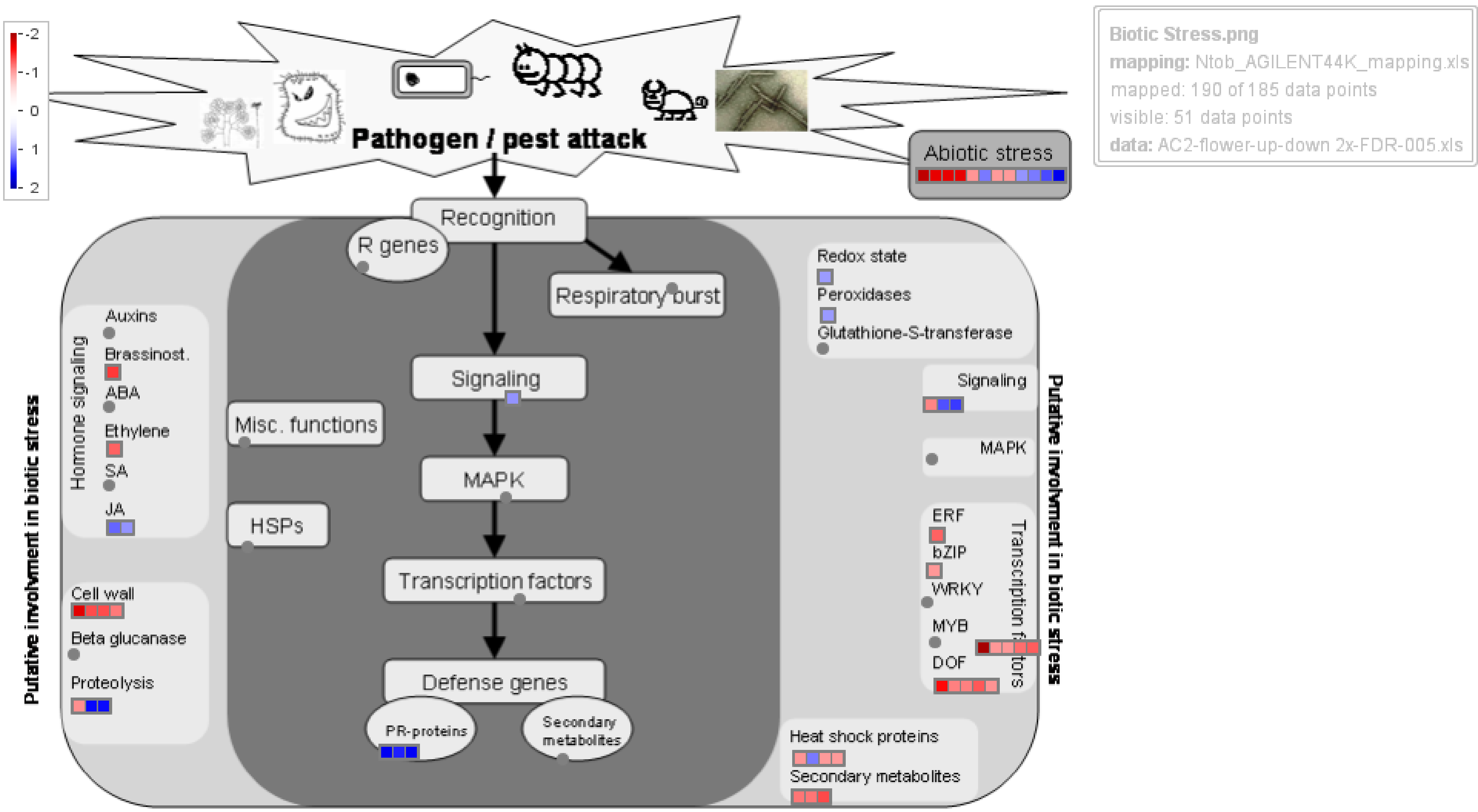

Biotic stress- HC-Pro leaf

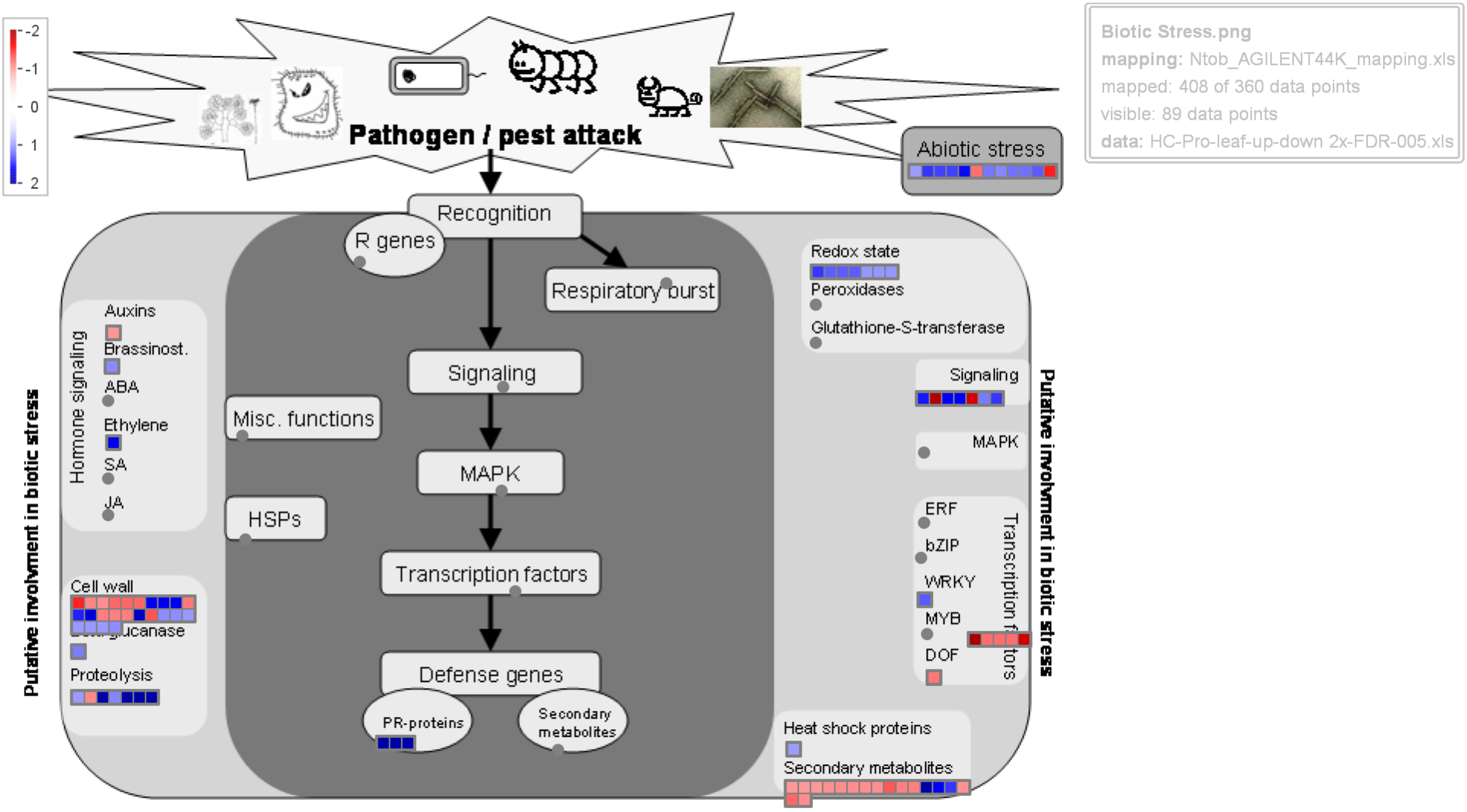

Biotic stress- HC-Pro flower

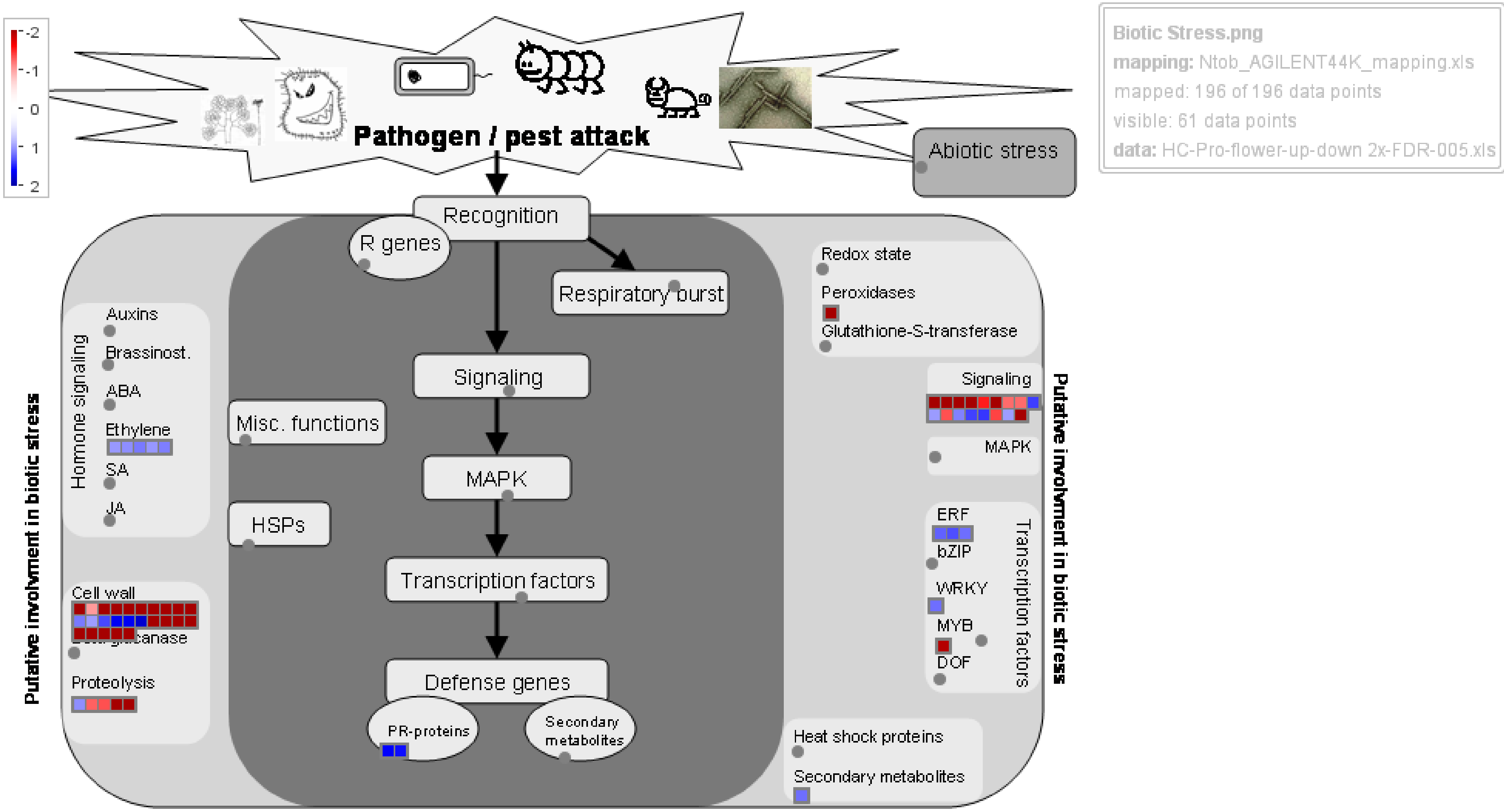

Supplement: Additional file 8 — Visual presentation of transcripts involved in biotic stress. Data consists of up or down regulated transcripts with p-values less than 0.05 (FDR) in leaf and flower samples expressing AC2 or HC-Pro RSS. [file 1471-2229-12-204-S8.pdf]
