# Supplementary material for: Expression of geminiviral AC2 RNA silencing suppressor changes sugar and jasmonate responsive gene expression in transgenic tobacco plants
Source: BMC Plant Biol. 2012 Nov 7;12:204. doi: 10.1186/1471-2229-12-204 (PMC3519546; doi:10.1186/1471-2229-12-204)
Supplement: Additional file 11 — PCR primers for RT-qPCR and methylation sensitive restriction enzyme amplified PCR. [file 1471-2229-12-204-S11.docx]

**Additional file 11. Primers used in RT-qPCR and methylation sensitive restriction enzyme PCR**

AC2; Primer pair for testing AC2 expression

LEFT PRIMER cactcacagacaagtcaagaagc

RIGHT PRIMER tggaagttgagaaaacatttgtg

SEQUENCE SIZE: 320

EB681684; Actin binding protein 1L; ABIL-1L

LEFT PRIMER AACTTCGGATTGATGGCTTG

RIGHT PRIMER TTAGCTTTCGAGCCTCCTGA

SEQUENCE SIZE: 188

X67159; Nicotiana tabacum Nicotiana tabacum pectate lyase mRNA

LEFT PRIMER TGGGAAAGGTACGCTATTGG

RIGHT PRIMER TCGCACCATTTTCCATATCA

SEQUENCE SIZE: 184

X65700, Nicotiana tabacum osmotin, AP-24 mRNA

LEFT PRIMER ATCGAGGTCCGAAACAACTG

RIGHT PRIMER AGCGTATTCAGCCAAGGTGT

SEQUENCE SIZE: 252

FG156808; Nicotiana tabacum P-rich protein NtEIG-C29

LEFT PRIMER CATCTGGCTTCCAGTGTCCT

RIGHT PRIMER CGTCCCCTAGAAAACCCTGT

SEQUENCE SIZE: 287

EB438380; Solanum lycopersicum Trypsin and protease inhibitor

LEFT PRIMER CCTAGTGCCACATCACGTTG

RIGHT PRIMER GCAATACATTGCTGGGAGTTT

SEQUENCE SIZE: 428

EH615198; Nicotiana tabacum nictaba (NT1)

LEFT PRIMER GGCTTCGATATTCGTCCAAA

RIGHT PRIMER TCACATGCACTTACAACAAGCTC

SEQUENCE SIZE: 211

FG156808; Nicotiana tabacum 1-D-deoxyxylulose 5-phosphate synthase

LEFT PRIMER CCTAGAGGAAATGGAGTTGGTG

RIGHT PRIMER GATGTCTGCAGCTCCCAAA

SEQUENCE SIZE: 210

NP917355; Nicotiana tabacum mRNA for ERF1

LEFT PRIMER CGGTTTAAATGAACCGGAAC

RIGHT PRIMER CATAGATCGAACCGGAAGGA

SEQUENCE SIZE: 387

X16077; Nicotiana tabacum mRNA for 18S rRNA

LEFT PRIMER GCGGATGTTGCTTTTAGGAC

RIGHT PRIMER GGTAAGTTTCCCCGTGTTGA

SEQUENCE SIZE 164
