# Supplementary material for: Expression of geminiviral AC2 RNA silencing suppressor changes sugar and jasmonate responsive gene expression in transgenic tobacco plants
Source: BMC Plant Biol. 2012 Nov 7;12:204. doi: 10.1186/1471-2229-12-204 (PMC3519546; doi:10.1186/1471-2229-12-204)
Supplement: Additional file 12 — Visual presentation of transcripts involved in cellular responses overview. Data consists of up or down regulated transcripts in leaf samples expressing AC2 or HC-Pro RSS. [file 1471-2229-12-204-S12.pdf]

RNA-ProteinSynthesis.xml Mapping: Ntob_AGILENT44K_mapping.xls

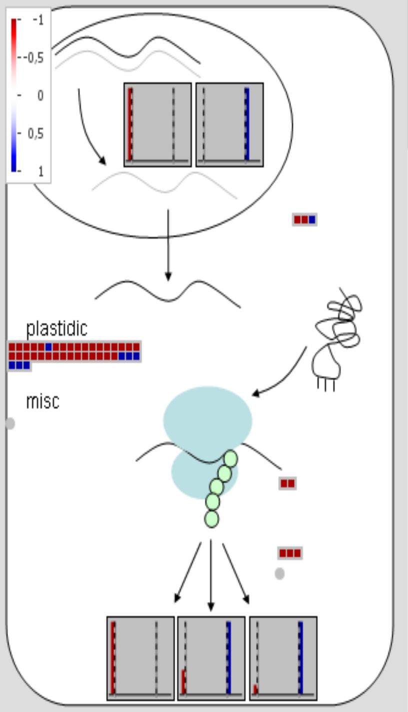

RNA-ProteinSynthesis.png
mapping: Ntob_AGILENT44K_mapping.xls
mapped: 736 of 680 data points
visible: 86 data points
data: AC2-leaf-up-down 2x-FDR-005.xls

RNA-Protein synthesis
AC2 leaf

Wilcoxon Rank Sum Test | InfoTable | Log

Correction: Benjamini Hochberg corre...

Bin		Elements	Probability	Present
29.2.1	protein.synthesis.ribosomal protein	33	1,56E-6	shown
29.2.4	protein.synthesis.elongation	3	0,18	shown
27.2	RNA.transcription	1	0,45	shown
29.5	protein.degradation	24	0,54	shown
29.3	protein.targeting	1	0,63	shown
29.2.3	protein.synthesis.initiation	2	0,64	shown
27.1	RNA.processing	1	0,67	shown
29.4	protein.postranslational modification	7	0,74	shown
29.1	protein.aa activation	3	0,75	shown
29.2.1.2	protein.synthesis.ribosomal protein.eukaryotic	26	6,05E-8	
29.2	protein.synthesis	38	6,05E-8	
29.2.1.2.2	protein.synthesis.ribosomal protein.eukaryotic.60S subunit	15	9,17E-6	
29	protein	72	2,85E-3	
17.5	hormone metabolism.ethylene	8	2,23E-2	
29.2.1.2.1	protein.synthesis.ribosomal protein.eukaryotic.40S subunit	11	2,23E-2	
29.5.11.4.3	protein.degradation.ubiquitin.E3.SCF	4	2,90E-2	
29.5.11.4.3.2	protein.degradation.ubiquitin.E3.SCF.FBOX	4	2,90E-2	
10	cell wall	10	5,56E-2	
20.1.7	stress.biotic.PR-proteins	3	7,04E-2	
20.1.7.6	stress.biotic.PR-proteins.proteinase inhibitors	3	7,04E-2	
20.1.7.6.1	stress.biotic.PR-proteins.proteinase inhibitors.trypsin inhibitor	3	7,04E-2	
17	hormone metabolism	26	7,98E-2	

RNA-ProteinSynthesis.xml Mapping: Ntob_AGILENT44K_mapping.xls

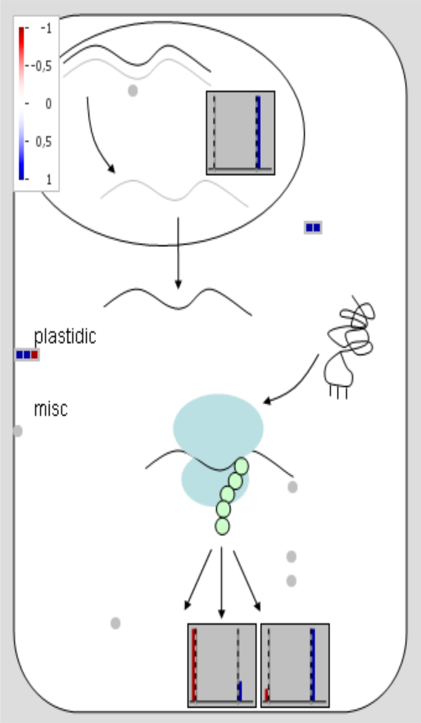

RNA-ProteinSynthesis.png
mapping: Ntob_AGILENT44K_mapping.xls
mapped: 408 of 360 data points
visible: 27 data points
data: HC-Pro-leaf-up-down 2x-FDR-005.xls

RNA-Protein synthesis
HC-Pro leaf

Wilcoxon Rank Sum Test | InfoTable | Log

Correction: Benjamini Hochberg corre...

Bin		Elements	Probability	Present
29.5	protein.degradation	7	0,23	shown
29.4	protein.postranslational modification	12	0,38	shown
29.1	protein.aa activation	2	0,56	shown
29.2.1	protein.synthesis.ribosomal protein	3	0,63	shown
27.1	RNA.processing	1	0,94	shown
29.5.11.4.3	protein.degradation.ubiquitin.E3.SCF	5	7,77E-2	
29.5.11.4.3.2	protein.degradation.ubiquitin.E3.SCF.FBOX	5	7,77E-2	
34	transport	8	7,77E-2	
20.1.7	stress.biotic.PR-proteins	3	7,77E-2	
20.1.7.6	stress.biotic.PR-proteins.proteinase inhibitors	3	7,77E-2	
20.1.7.6.1	stress.biotic.PR-proteins.proteinase inhibitors.trypsin inhibitor	3	7,77E-2	
26.18	misc.invertase/pectin methylesterase inhibitor family protein	6	7,77E-2	
34.99	transport.misc	6	0,15	
27.3.26	RNA.regulation of transcription.MYB-related transcription factor fa...	4	0,21	
20.1	stress.biotic	5	0,23	
29.5.11	protein.degradation.ubiquitin	7	0,23	
29.5.11.4	protein.degradation.ubiquitin.E3	7	0,23	
27.3.66	RNA.regulation of transcription.Psudo ARR transcription factor family	2	0,27	
14	S-assimilation	2	0,27	
14.2	S-assimilation.APR	2	0,27	
3.4.3	minor CHO metabolism.myo-inositol.InsP Synthases	2	0,29	
35	not assigned	163	0,29	

Cellular_response_overview.xml Mapping: Ntob_AGILENT44K_mapping.xls

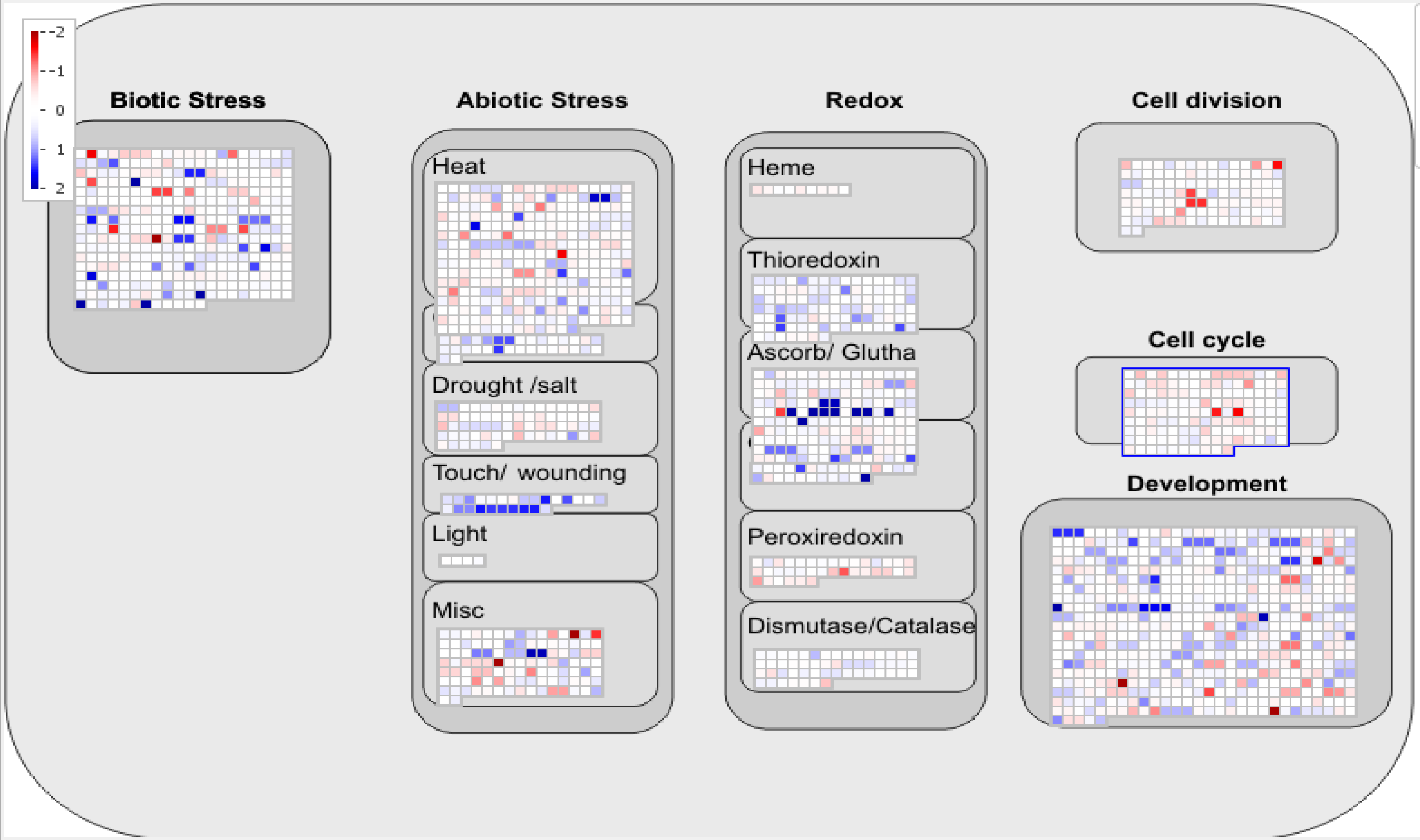

Cellular_response_overview.png
mapping: Ntob_AGILENT44K_mapping.xls
mapped: 39515 of 43759 data points
visible: 2023 data points
data: AC2- leaf-all-duplications removed.xls

Cellular responses

AC2-leaf

Cell cycle

Wilcoxon Rank Sum Test | InfoTable | Log

BinCode	BinName	id	type	description	AC2- leaf-...
31.3	cell.cycle	a_95_p294588	Transcript	weakly similar to (189) AT4G34160 | Symbols: CYCD3;1, CYCD3 | CYCD3;1 (CYCLIN D3;1); cyclin-dependent protein kinase regulator/ pr...	-1.63
31.3	cell.cycle	a_95_p268576	Transcript	weakly similar to (145) AT4G34160 | Symbols: CYCD3;1, CYCD3 | CYCD3;1 (CYCLIN D3;1); cyclin-dependent protein kinase regulator/ pr...	-1.533
31.3	cell.cycle	a_95_p076905	Transcript	weakly similar to (108) AT3G11520 | Symbols: CYCB1;3, CYC2 | CYCB1;3 (CYCLIN B1;3); cyclin-dependent protein kinase regulator | chr3...	-1.051
31.3	cell.cycle	a_95_p217622	Transcript	moderately similar to (480) AT1G44110 | Symbols: CYCA1;1 | CYCA1;1 (Cyclin A1;1); cyclin-dependent protein kinase regulator | chr1:16...	-0.809
31.3	cell.cycle	a_95_p008451	Transcript	moderately similar to (394) AT5G06150 | Symbols: CYC1BAT, CYCB1;2 | CYC1BAT; cyclin-dependent protein kinase regulator | chr5:1859...	-0.794
31.3	cell.cycle	a_95_p028066	Transcript	moderately similar to (354) AT5G43080 | Symbols: CYCA3;1 | CYCA3;1 (Cyclin A3;1); cyclin-dependent protein kinase regulator | chr5:17...	-0.786
31.3	cell.cycle	a_95_p020846	Transcript	moderately similar to (297) AT5G67260 | Symbols: CYCD3;2 | CYCD3;2 (CYCLIN D3;2); cyclin-dependent protein kinase | chr5:26836313-...	-0.757
31.3	cell.cycle	a_95_p020846	Transcript	moderately similar to (299) AT5G67260 | Symbols: CYCD3;2 | CYCD3;2 (CYCLIN D3;2); cyclin-dependent protein kinase | chr5:26836313-...	-0.757
31.3	cell.cycle	a_95_p020846	Transcript	moderately similar to (300) AT5G67260 | Symbols: CYCD3;2 | CYCD3;2 (CYCLIN D3;2); cyclin-dependent protein kinase | chr5:26836313-...	-0.757
31.3	cell.cycle	a_95_p010676	Transcript	moderately similar to (393) AT5G06150 | Symbols: CYC1BAT, CYCB1;2 | CYC1BAT; cyclin-dependent protein kinase regulator | chr5:1859...	-0.724
31.3	cell.cycle	a_95_p118888	Transcript	weakly similar to (187) AT2G26760 | Symbols: CYCB1;4 | CYCB1;4 (Cyclin B1;4); cyclin-dependent protein kinase regulator | chr2:114015...	-0.72
31.3.1	cell.cycle.pe...	a_95_p193967	Transcript	moderately similar to (301) AT5G13120 | Symbols: | peptidyl-prolyl cis-trans isomerase cyclophilin-type family protein | chr5:4162714-41...	-0.71
31.3.1	cell.cycle.pe...	a_95_p095653	Transcript	weakly similar to (164) AT1G26550 | Symbols: | peptidyl-prolyl cis-trans isomerase PPIC-type family protein | chr1:9171800-9172716 FO...	-0.681
31.3	cell.cycle	a_95_p023061	Transcript	moderately similar to (222) AT3G21870 | Symbols: CYCP2;1 | CYCP2;1 (cyclin p2;1); cyclin-dependent protein kinase | chr3:7703927-770...	-0.666
31.3.1	cell.cycle.pe...	a_95_p272466	Transcript	weakly similar to (191) AT1G74070 | Symbols: | peptidyl-prolyl cis-trans isomerase cyclophilin-type family protein | chr1:27851749-27852...	-0.649
31.3.1	cell.cycle.pe...	a_95_p043141	Transcript	moderately similar to (301) AT5G13120 | Symbols: | peptidyl-prolyl cis-trans isomerase cyclophilin-type family protein | chr5:4162714-41...	-0.622
31.3.1	cell.cycle.pe...	a_95_p043141	Transcript	moderately similar to (301) AT5G13120 | Symbols: | peptidyl-prolyl cis-trans isomerase cyclophilin-type family protein | chr5:4162714-41...	-0.622

Cellular_response_overview.xml Mapping: Ntob_AGILENT44K_mapping.xls

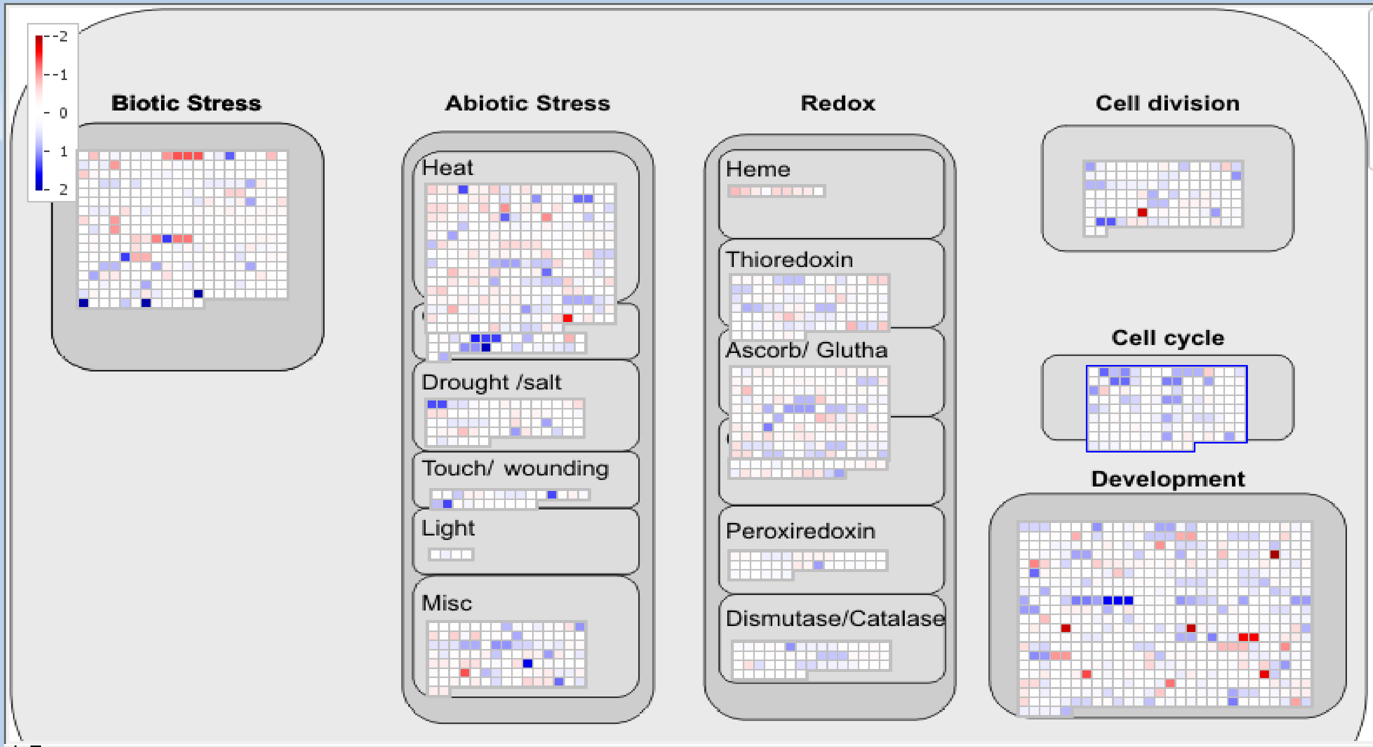

Cellular_response_overview.png
mapping: Ntob_AGILENT44K_mapping.xls
mapped: 39515 of 43759 data points
visible: 2023 data points
data: HC-Pro- leaf-all-duplications removed.xls

Cellular responses
HC-Pro leaf
Cell cycle

Wilcoxon Rank Sum Test | InfoTable | Log

BinCode	BinName	id	type	description	HC-Pro- leaf-...
31.3	cell.cycle	a_95_p150867	Transcript	weakly similar to (134) AT5G26850 | Symbols: | INVOLVED IN: biological_process unknown; LOCATED IN: chloroplast; EXPRESSED IN: 23 ...	0.614
31.3	cell.cycle	a_95_p209127	Transcript	moderately similar to (407) AT1G80370 | Symbols: CYCA2;4 | CYCA2;4 (Cyclin A2;4); cyclin-dependent protein kinase regulator | chr1:302...	0.65
31.3	cell.cycle	a_95_p237869	Transcript	moderately similar to (306) AT5G67260 | Symbols: CYCD3;2 | CYCD3;2 (CYCLIN D3;2); cyclin-dependent protein kinase | chr5:26836313-2...	0.773
31.3	cell.cycle	a_95_p208287	Transcript	moderately similar to (239) AT3G05330 | Symbols: ATN, ATTAN | ATN (TANGLED) | chr3:1519162-1521159 REVERSEmoderately similar to ...	0.783
31.3	cell.cycle	a_95_p010506	Transcript	moderately similar to (394) AT2G26760 | Symbols: CYCB1;4 | CYCB1;4 (Cyclin B1;4); cyclin-dependent protein kinase regulator | chr2:114...	0.82
31.3	cell.cycle	a_95_p020846	Transcript	moderately similar to (297) AT5G67260 | Symbols: CYCD3;2 | CYCD3;2 (CYCLIN D3;2); cyclin-dependent protein kinase | chr5:26836313-2...	0.884
31.3	cell.cycle	a_95_p020846	Transcript	moderately similar to (299) AT5G67260 | Symbols: CYCD3;2 | CYCD3;2 (CYCLIN D3;2); cyclin-dependent protein kinase | chr5:26836313-2...	0.884
31.3	cell.cycle	a_95_p020846	Transcript	moderately similar to (300) AT5G67260 | Symbols: CYCD3;2 | CYCD3;2 (CYCLIN D3;2); cyclin-dependent protein kinase | chr5:26836313-2...	0.884
31.3.1	cell.cycle.pe...	a_95_p027476	Transcript	moderately similar to (244) AT4G32420 | Symbols: | peptidyl-prolyl cis-trans isomerase cyclophilin-type family protein | chr4:15647550-15...	0.92
31.3.1	cell.cycle.pe...	a_95_p180872	Transcript	moderately similar to (258) AT2G16600 | Symbols: ROC3 | ROC3; peptidyl-prolyl cis-trans isomerase | chr2:7200862-7201383 FORWARD...	0.966
31.3.1	cell.cycle.pe...	a_95_p207922	Transcript	moderately similar to (244) AT4G32420 | Symbols: | peptidyl-prolyl cis-trans isomerase cyclophilin-type family protein | chr4:15647550-15...	0.971
31.3	cell.cycle	a_95_p076905	Transcript	weakly similar to (108) AT3G11520 | Symbols: CYCB1;3, CYC2 | CYCB1;3 (CYCLIN B1;3); cyclin-dependent protein kinase regulator | chr3:...	0.975
31.3	cell.cycle	a_95_p217677	Transcript	moderately similar to (306) AT5G67260 | Symbols: CYCD3;2 | CYCD3;2 (CYCLIN D3;2); cyclin-dependent protein kinase | chr5:26836313-2...	1.0
31.3	cell.cycle	a_95_p118888	Transcript	weakly similar to (187) AT2G26760 | Symbols: CYCB1;4 | CYCB1;4 (Cyclin B1;4); cyclin-dependent protein kinase regulator | chr2:1140155...	1.019
31.3	cell.cycle	a_95_p217622	Transcript	moderately similar to (480) AT1G44110 | Symbols: CYCA1;1 | CYCA1;1 (Cyclin A1;1); cyclin-dependent protein kinase regulator | chr1:167...	1.044
31.3	cell.cycle	a_95_p010676	Transcript	moderately similar to (393) AT5G06150 | Symbols: CYC1BAT, CYCB1;2 | CYC1BAT; cyclin-dependent protein kinase regulator | chr5:18595...	1.09
31.3	cell.cycle	a_95_p048216	Transcript	moderately similar to (394) AT5G06150 | Symbols: CYC1BAT, CYCB1;2 | CYC1BAT; cyclin-dependent protein kinase regulator | chr5:18595...	1.146
31.3	cell.cycle	a_95_p048216	Transcript	weakly similar to (118) AT5G06150 | Symbols: CYC1BAT, CYCB1;2 | CYC1BAT; cyclin-dependent protein kinase regulator | chr5:1859542-1...	1.146
31.3	cell.cycle	a_95_p034174	Transcript	moderately similar to (394) AT5G06150 | Symbols: CYC1BAT, CYCB1;2 | CYC1BAT; cyclin-dependent protein kinase regulator | chr5:18595...	1.238
31.3	cell.cycle	a_95_p034174	Transcript	weakly similar to (118) AT5G06150 | Symbols: CYC1BAT, CYCB1;2 | CYC1BAT; cyclin-dependent protein kinase regulator | chr5:1859542-1...	1.238
31.3	cell.cycle	a_95_p008451	Transcript	moderately similar to (394) AT5G06150 | Symbols: CYC1BAT, CYCB1;2 | CYC1BAT; cyclin-dependent protein kinase regulator | chr5:18595...	1.257
